# Wearable camera-derived microenvironments in relation to personal exposure to PM_2.5_

**DOI:** 10.1016/j.envint.2018.05.021

**Published:** 2018-08

**Authors:** Maëlle Salmon, Carles Milà, Santhi Bhogadi, Srivalli Addanki, Pavitra Madhira, Niharika Muddepaka, Amaravathi Mora, Margaux Sanchez, Sanjay Kinra, V. Sreekanth, Aiden Doherty, Julian D. Marshall, Cathryn Tonne

**Affiliations:** a ISGlobal, Universitat Pompeu Fabra, CIBER Epidemiología y Salud Pública, Barcelona, Spain.; b Public Health Foundation of India, New Delhi, India; c Department of Civil and Environmental Engineering, University of Washington, Seattle, WA, USA; d Department of Non-communicable Disease Epidemiology, London School of Hygiene and Tropical Medicine, London, UK; e Big Data Institute, Nuffield Department of Population Health, University of Oxford, Oxford, UK

**Keywords:** Personal exposure, Particulate matter, Microenvironments, Time-activity, Wearable cameras, India

## Abstract

Data regarding which microenvironments drive exposure to air pollution in low and middle income countries are scarce. Our objective was to identify sources of time-resolved personal PM_2.5_ exposure in peri-urban India using wearable camera-derived microenvironmental information. We conducted a panel study with up to 6 repeated non-consecutive 24 h measurements on 45 participants (186 participant-days). Camera images were manually annotated to derive visual concepts indicative of microenvironments and activities. Men had slightly higher daily mean PM_2.5_ exposure (43 μg/m^3^) compared to women (39 μg/m^3^). Cameras helped identify that men also had higher exposures when near a biomass cooking unit (mean (sd) μg/m^3^: 119 (383) for men vs 83 (196) for women) and presence in the kitchen (133 (311) for men vs 48 (94) for women). Visual concepts associated in regression analysis with higher 5-minute PM_2.5_ for both sexes included: smoking (+93% (95% confidence interval: 63%, 129%) in men, +29% (95% CI: 2%, 63%) in women), biomass cooking unit (+57% (95% CI: 28%, 93%) in men, +69% (95% CI: 48%, 93%) in women), visible flame or smoke (+90% (95% CI: 48%, 144%) in men, +39% (95% CI: 6%, 83%) in women), and presence in the kitchen (+49% (95% CI: 27%, 75%) in men, +14% (95% CI: 7%, 20%) in women). Our results indicate wearable cameras can provide objective, high time-resolution microenvironmental data useful for identifying peak exposures and providing insights not evident using standard self-reported time-activity.

## Highlights

• Paired time-resolved wearable camera activity and personal PM_2.5_ exposure data
• Men had higher PM_2.5_ exposure than women in most microenvironments.
• Smoking and cooking identified by cameras associated with increased PM_2.5_.
• Wearable cameras can be valuable tool for identifying drivers of personal exposure.

## 1 Introduction

Fine particulate matter (particle matter with diameter of 2.5 μm or less, PM_2.5_), has diverse adverse health effects (WHO Regional Office for Europe, 2013) and is associated with a large public health burden globally (8% of global deaths) (Cohen et al., 2017; Gakidou et al., 2017). Data on exposure to particulate matter and the influence of specific microenvironments on exposure in low and middle income countries such as India are scarce (Pant et al., 2016). Current knowledge indicates that the sources of exposure to PM_2.5_ in India are diverse (Pant et al., 2016) and include occupational (Semple et al., 2008), household (e.g. solid-fuel cooking) (Andresen et al., 2005; Balakrishnan et al., 2011), and travel-related sources (Pant et al., 2017). However, most studies have focused on a single source of exposure, providing little insight into the relative contribution to personal exposure of different microenvironments and sources.

Developing effective interventions to reduce PM_2.5_ exposure requires understanding which sources drive personal exposure, which is influenced by concentrations in each microenvironment and time spent in that microenvironment (Duan, 1982)_._ Microenvironmental information is often derived from time-activity diaries or questionnaires (Buonanno et al., 2014; Deffner et al., 2016; Gu et al., 2015; Gurung and Bell, 2012; Steinle et al., 2015), which rely on participants' motivation and memory and are therefore potentially biased. Moreover, these data typically provide coarse temporal resolution that can be improved only with significant increase in the burden for participants. Microenvironments are only partially measured via Global Positioning System (GPS) data, which may not capture information on different activities performed at the same location. There is therefore a need for tools collecting more objective, precise, and time-resolved microenvironmental information in personal exposure studies.

Wearable camera technology has been recently used in health studies to assess physical activity, nutrition, and the environment, e.g., amount of green space (Doherty et al., 2013a; Mavoa et al., 2013; Oliver et al., 2013). To our knowledge this technology has not previously been used to identify drivers of exposure to air pollution, nor worn with time-resolved environmental monitors.

Our study represents the first use of wearable cameras in combination with a personal PM_2.5_ monitor; we employ high temporal resolution data in a panel study with repeated sampling per participant. Our objective was to identify sources of time-resolved personal PM_2.5_ exposure using wearable camera-derived microenvironmental information in a sample of the general population in peri-urban India.

## 2 Material and methods

### 2.1 Study design

We used data from the Cardiovascular Health effects of Air pollution in Telangana, India (CHAI) project (Tonne et al., 2017), nested in the Andhra Pradesh Children and Parents Study (APCAPS). APCAPS is an intergenerational cohort of about 6000 participants living near Hyderabad, Telangana, India (Kinra et al., 2014). The study population lived in 28 villages spread over a 22 km × 35 km region southeast of Hyderabad; population per village ranged from 187 to 5065 households. CHAI was approved by the Ethics Committees of Parc de Salut MAR (Barcelona, Spain), the Indian Institute of Public Health-Hyderabad (Hyderabad, India), and the National Institute of Nutrition (Hyderabad, India).

CHAI recruited a random sample of 538 APCAPS participants for personal monitoring, including a subsample of 60 participants willing to participate in an intensive panel study. Panel study measurements included up to six 24-hour non-consecutive sessions with a PM_2.5_ monitor and a wearable camera. Sessions were performed between May 2015 and February 2016. Sessions typically started at 8 am when a field worker set up the devices at the participant's home and ended 24 h later when a field worker recovered the devices. Sessions included weekends and weekdays; starting times were planned to avoid disrupting participants' usual activities. Field workers reported that participants adapted quickly to wearing the devices and had few complaints.

### 2.2 Wearable camera data

**Fig. 1:**
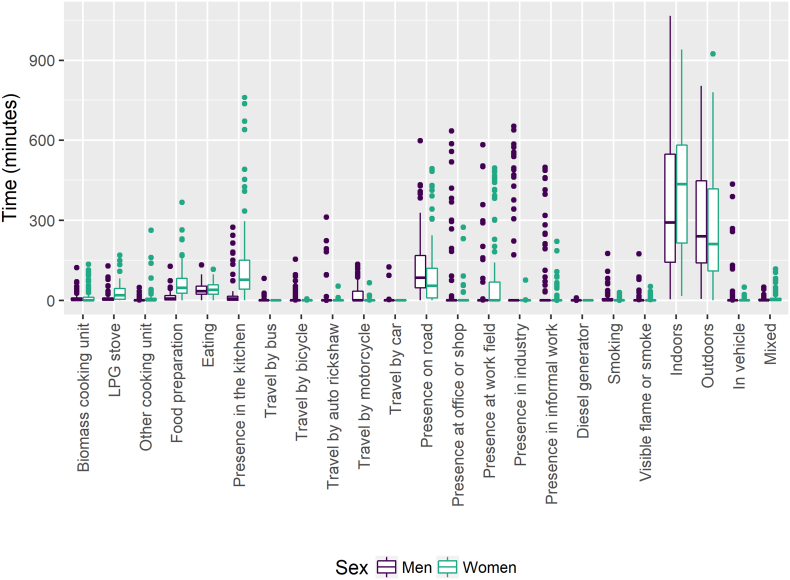
Minutes per day according to visual concepts derived from wearable camera. Data are stratified by sex. Whiskers correspond to 1.5*interquartile range; data beyond the whiskers are considered outliers and plotted as points.

Participants wore a wearable camera (Autographer, OMG Life, Oxford, UK) via a neck-worn lanyard on their chest. The camera took a photograph approximately every 35 s (S-Fig. 1). Participants were asked to turn cameras off at night due to limited battery life (approximately 10 h), and to remove or turn them off whenever they felt uncomfortable wearing them, according to standard ethics procedures (Kelly et al., 2013).

Trained staff categorized visual concepts to identify microenvironments and activities in each image. Visual concepts corresponded to 5 non-exclusive categories identified a priori as being potentially relevant for PM_2.5_ exposure: travel, occupation, cooking, indoor/outdoor location, and presence of other types of combustion (annotation protocol in supporting information). Inter-rater agreement between annotators was computed using Cohen's Kappa (Cohen, 1960) as in other scientific studies using Autographers (Doherty et al., 2013b; Kelly et al., 2012; Nguyen et al., 2009). Annotators were required to achieve inter-rater agreement of Kappa = 0.85 in each category using a training dataset before being allowed to annotate main study data. Duplicate annotation by a second annotator was performed on 45 participant-days (25% of the total sample, 45 unique participants) to assess inter-rater agreement during annotation of the main study data.

**Fig. 2:**
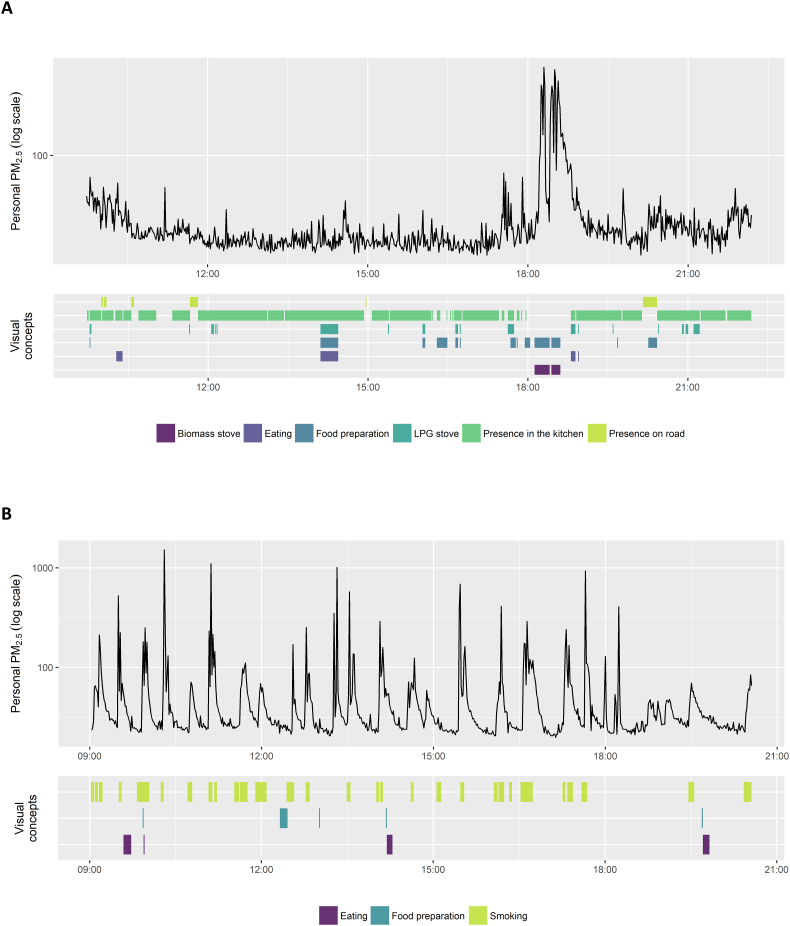
Time series of personal PM_2.5_ exposure with simultaneous wearable camera derived visual concept for a A) female and B) male participant.

Details of the aggregation of visual concepts derived from the wearable cameras for 5-min intervals are in the SI (S-Fig. 2). Briefly, the annotation process generated a dataset of Boolean values by photograph in which annotation = TRUE indicates that a photograph had a given annotation. We aggregated this dataset to generate values by minute (Boolean, annotation=TRUE indicates the annotation was present at some point during that minute) and subsequently by 5 minute intervals (continuous, indicating the proportion of the 5 minute interval for which the code was present e.g. 1 when present in all of them). The 5-min aggregated dataset was used in regression analysis.

### 2.3 Self-reported time-activity

A questionnaire was administered by a field worker after each monitoring session. Field workers interviewed participants to complete an hourly time-activity questionnaire reporting main locations (e.g., indoor at home, outdoor in village) and main activities (e.g., cooking, sleeping, working) for the prior 24 h. This standard questionnaire, developed at Sri Ramachandra University, has been used in previous studies involving PM_2.5_ measurements (Balakrishnan et al., 2004). Each hour could be associated with 1 or 2 activity-location tuple(s): participants could mention one or two activity(ies) per hour, but were not asked about the proportion of the hour spent in each activity. Questionnaire data was collected on paper and manually entered into a database via a custom interface.

### 2.4 Personal PM_2.5_ exposure data

**Fig. 3:**
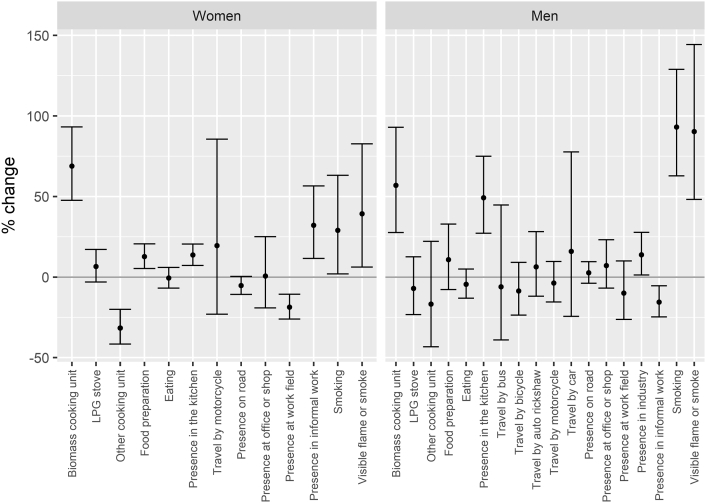
Percent change and 95% confidence intervals in 5-minute average PM_2.5_ associated with wearable camera derived visual concepts. Regression coefficients are mutually adjusted.

Participants wore the RTI MicroPEM v 3.2A. (MicroPEM, RTI International, Research Triangle Park, NC 27709, USA) PM_2.5_ monitor near the breathing zone. We used four MicroPEM devices throughout the field work. Prior to data collection, three MicroPEMs were collocated indoors, and we observed good agreement between devices (median ratios in pairwise comparisons were 0.99, 1.07, and 1.06; see S-Fig. 3). Further details on collocation results are included in S-Table 1. We followed the standard operating procedure provided by the manufacturer (RTI International, USA), which involved cleaning the inlets and impactor, adjusting the zero offset with HEPA filter attached, calibrating the pump flow rate using an external mass flowmeter (TSI 4140), and calibrating the temperature and humidity sensors before each deployment in the field. Post-sampling flow was measured to detect drift in the flow rate. We discarded output files (i.e. one person-day of measurements) if the difference between pre and post-sampling flow rate was >20%. We manually checked parameters (e.g., inlet and orifice pressures) to identify leakage of air in the instrument or other malfunctions. We also omitted files with abrupt baseline shifts (e.g., attributable to contamination of the optics during measurements).

We used the R package rtimicropem (Salmon et al., 2017) for MicroPEM data processing. We identified outliers in the PM_2.5_ time-series using the R-function forecast::tsoutlier and removed these data points before applying temperature sensitivity and gravimetric corrections. We identified temperature sensitivity in some of the MicroPEM devices above 30 °C when conducting experiments with HEPA filters attached to each device during measurements over a range of temperatures. We derived device-specific correction equations that were applied to each output file. MicroPEMs can accommodate a 27 mm Teflon filter for gravimetric correction of the time-series based on the mass collected on the filter paper. We identified small holes in most of the Teflon filter papers and therefore did not rely on the MicroPEM gravimetric mass measurements for correcting the nephelometer measurements. Instead, we used measurements from SKC gravimetric samplers (a SKC pump which drew air through a sharp cut cyclone attached to a cassette containing a 37 mm filter) worn simultaneously with the MicroPEM to derive a linear regression equation to correct nephelometer values. Further details of the temperature and gravimetric correction are included in the R package documentation (Salmon, 2017a). The MicroPEM sampling rate was every 10 s; data were processed to obtain one-minute averages. Gaps in the series smaller than 5 min were interpolated; gaps >5 min were left as missing.

### 2.5 Analyses

Although data coverage during the 24-hour session from the MicroPEM was relatively complete, wearable camera data was limited during nighttime hours (S-Fig. 4), as per instructions to participants. Analyses using the wearable camera-derived microenvironmental information mostly cover the 7 am–8 pm interval. All analyses were stratified by sex, which was strongly associated with activity patterns in our study population (Sanchez et al., 2017).

We calculated average PM_2.5_ concentrations for minutes corresponding with a visual concept from the wearable camera data. Because more than one visual concept can occur for a given photograph, we used regression models to estimate the independent effect of a visual concept on 5-min average PM_2.5_, accounting for temporal autocorrelation and the repeated measures design. This analysis allowed us to identify visual concepts with statistically significant associations with 5-min average PM_2.5_ exposure. Models were fit using Gamma family generalized linear models, including a random intercept for each participant-day to account for the repeated sampling and an auto-regressive correlation structure of lag 1 within participant-day to take into account temporal autocorrelation in the time series. For these models, the overall intercept corresponds to the 5-minute average PM_2.5_ when no visual concept was present, and the coefficient corresponds to the PM_2.5_ concentration increment for a 5-minute interval in which 100% of the images contained the visual concept. To improve model fit, we excluded data for infrequent activities (occurring in <100 photographs). Visual concepts related to location were omitted in the regression model because of collinearity (i.e., many activities only occur in a single location). We report associations as percent change in exposure associated with a given visual concept: [exp(β_i_) − 1] ∗ 100 where β_i_ is the regression coefficient for the visual concept. We conducted similar descriptive and regression analyses for the self-reported activity data in relation to PM_2.5_. The regression model differed slightly for hourly PM_2.5_ in that it included random intercepts for participant IDs and person-days; here, the intercept corresponded to sleeping. Data preparation and statistical analyses were conducted in R version 3.3.1 (R Core Team, 2016) using several packages (Boettiger and Jones, 2017; Clifford, 2017; Garnier, 2017; Grolemund and Wickham, 2011; Henry and Wickham, 2017; Lawrence et al., 2013; Robinson, 2016, Robinson, 2017; Salmon, 2017b; Salmon et al., 2017; Wickham, 2016a, Wickham, 2016b, Wickham, 2017a, Wickham, 2017b; Wickham et al., 2017; Wilke, 2016; Wood, 2006; Xie, 2015).

## 3 Results

Main characteristics of the study population are presented in Table 1. The mean age was 44 years (sd = 14). Women were older, and more likely to be illiterate and to have unskilled manual occupation than men. Among men, 27% reported current active smoking; no women reported active smoking. We excluded 24 participant-days (11% of the raw data) because of non-compliance or lack of wearable camera or MicroPEM data. Excluded men were younger, less often married, and smoked less than included men while excluded women were less likely to be in agricultural-related occupations than included women (S-Table 2). However, there were no statistically significant differences between included participants and a random sample of the APCAPS cohort (S-Table 2) indicating good representativeness of the data to the APCAPS population. Overall 286,302 photographs were collected for 186 participant-days (45 participants) with both Autographer and MicroPEM data, with a median of 1330 photographs per participant-day (median wear-time: 12.3 h per participant-day). The mean (sd) number of monitoring sessions by participant was 4.1 (1.5); 9% of participants had only one session.

**Table 1:** Characteristics of study population.

	All	Women	Men
N	45	23	22
Age (years), m (sd)	44 (13.8)	48 (8.8)	40 (16.9)
Min–max	21–65	29–62	21–65
Number of sessions, m (sd)	4.1 (1.5)	4.1 (1.4)	4.1 (1.6)
Only 1 session, n (%)	4 (9)	2 (9)	2 (9)
Marital status, married, n (%)	31 (69)	17 (74)	14 (64)
Education level, illiterate, n (%)	24 (53)	18 (78)	6 (27)
Current smoker, n (%)	6 (13)	0 (0)	6 (27)
Primary occupation, n (%)			
Unemployed	4 (9)	2 (9)	2 (9)
Unskilled manual	24 (53)	16 (70)	8 (36)
Semi-skilled manual	6 (13)	3 (13)	3 (14)
Skilled manual	9 (20)	1 (4)	8 (36)
Non manual	2 (4)	1 (4)	1 (5)
Agriculture-related occupation, n (%)	22 (49)	16 (70)	6 (27)
Body mass index (kg/m^2^), n (%)			
<18.5	12 (27)	6 (26)	6 (27)
18.5–23.0	21 (47)	9 (39)	12 (55)
≥23.0	11 (24)	8 (35)	3 (14)
Primary fuel use, n (%)^a^			
Biomass	15 (33)	3 (13)	12 (55)
Liquefied petroleum gas (LPG)	39 (87)	22 (96)	17 (77)
Others	9 (20)	5 (22)	4 (18)

a More than one primary fuel type is possible.

Camera annotation inter-rater agreement values (S-Table 3) reflected almost perfect agreement, except for the relatively infrequent travel by auto-rickshaw visual concept for which the Kappa was 0.61, reflecting fair agreement. No Kappa could be computed for travel by bus because the visual concept did not occur in the duplicate annotation set.

For women, the most frequent visual concepts were presence in the kitchen (median time spent in that visual concept = 77 min/d), presence on road (55 min/d) and food preparation (48 min/d) (Fig. 1, S-Table 4). The most frequent visual concepts for men were presence on road (85 min/d) and eating (34 min/d), although some men also spent several hours in offices or shops and industry (Fig. 1, S-Table 4). By comparison, when considering self-reported time activity information (S-Table 5) covering the full 24-hour period, participants spent the most time sedentary, sleeping, and working. In self-report data, women spent a median of 3 h/d doing household chores and 2 h/d cooking and men spent 6 h/d working and 0 h/d cooking.

Overall PM_2.5_ exposures (24-h average including time periods with and without presence of visual concepts) were 39.3 μg/m^3^ for women and 42.8 μg/m^3^ for men. Average PM_2.5_ per visual concept varied from 32 to 84 μg/m^3^ for women and 37 to 138 μg/m^3^ for men (Table 2, S-Fig. 5). With few exceptions (e.g., presence in informal work, travel by bicycle), average exposure was higher for men than women for a given visual concept (Table 2), including some cooking-related activities: biomass cooking unit, food preparation, and presence in the kitchen; however, time linked to those cooking-related visual concepts was smaller for men than for women. Measured PM_2.5_ according to self-reported activity similarly showed that men often had higher exposures for a given self-reported activity (S-Table 6).

**Table 2:** Mean and median 1-minute averaged PM_2.5_ exposure (μg/m^3^) and frequency (minutes) according to visual concept by sex.

	Women: mean (sd)	Median	Total minutes	Men: mean (sd)	Median	Total minutes
Cooking						
Biomass cooking unit	84 (196)	34	1129	119 (384)	384	857
LPG stove	53 (136)	35	2659	91 (213)	213	1260
Other cooking unit	44 (50)	38	935	42 (31)	31	268
Food preparation	60 (130)	36	5691	99 (311)	311	1214
Eating	39 (42)	32	3975	47 (73)	73	3665
Presence in the kitchen	48 (94)	34	12,139	133 (311)	311	2196

Travel						
Travel by bus	NA	NA	0	46 (23)	23	181
Travel by bicycle	48 (15)	50	13	41 (28)	28	983
Travel by auto rickshaw	36 (14)	33	65	42 (34)	34	1212
Travel by motorcycle	39 (33)	32	86	37 (42)	42	2084
Travel by car	NA	NA	0	48 (38)	38	229
Presence on road	39 (57)	31	9023	47 (154)	154	11,376

Occupation						
Presence at office or shop	39 (13)	36	718	49 (58)	58	4699
Presence at work field	32 (11)	30	8384	38 (20)	20	1920
Presence in industry	34 (13)	30	78	54 (145)	145	9394
Presence in informal work	44 (72)	33	1000	39 (62)	62	5519

Presence of non-cooking combustion						
Diesel generator	NA	NA	0	45 (26)	26	21
Smoking^a^	49 (32)	36	191	104 (217)	217	800
Visible flame or smoke	71 (173)	31	226	138 (416)	416	631

Location						
Indoors	40 (55)	32	38,833	51 (112)	112	29,791
In vehicle	36 (15)	33	60	41 (32)	32	2158
Mixed	37 (29)	32	972	57 (79)	79	450

a Includes active and passive smoking.

Illustrative examples of personal exposure time series with simultaneous wearable camera derived data for one female and one male participant are presented in Fig. 2. According to the wearable camera data, the female participant spent the majority of the monitoring period in the kitchen, with a few short periods outside on the road (Fig. 2A). A LPG stove was visible in photos at several times throughout the day, including the period around 14:30 that corresponded with food preparation and eating, most likely the mid-day meal. A biomass stove was visible in photos around 18:30, during which food preparation and eating were also visible, suggesting the evening meal. While periods with a visible LPG stove corresponded with no or only moderate short-term increases in personal PM_2.5_, the biomass stove corresponded with a large increase in personal PM_2.5_. For the male participant, short-term increases in exposure had good temporal correspondence with photos containing a cigarette in the hand or mouth of the participant or another individual (Fig. 2B).

In regression analyses smoking was statistically significantly associated with higher 5-min average PM_2.5_ for both sexes, although the effect was more pronounced for men. Exposure was 93% higher (95% Confidence Interval (CI): 63%, 129%) when smoking was present compared to not present in the wearable camera images among men and 29% higher (95% CI: 2%, 63%) for women (Fig. 3). This could reflect differences in exposure from active vs passive smoking between men and women and proximity to cigarette smoke when in the presence of cigarettes. No women reported being current smokers (Table 1). In addition to smoking, several other visual concepts were significantly associated with increased 5-min PM_2.5_ exposure among women including: biomass cooking unit (69% (95% CI: 48%, 93%)); visible flame or smoke (39% (95% CI: 6%, 83%)), and presence in the kitchen (14% (95% CI: 7%, 20%)). However, presence of other cooking unit (−31% (95% CI: −41%, −20%)) and presence at work in an agricultural field (−19% (95% CI: −26%, −11%)) were significantly associated with lower exposures compared to intervals when these visual concepts were not present (Fig. 3). Among men, visual concepts significantly associated with higher 5-minute PM_2.5_ exposure included visible flame or smoke (90% higher PM2.5 compared to intervals without visible flame or smoke present (95% CI: 48%, 144%)), biomass cooking unit (57% (95% CI: 28%, 93%)), presence in the kitchen (49% (95% CI: 27%, 75%)), and presence in industry (14% (95% CI: 1%, 28%)). In contrast to women, presence in informal work for men was associated with lower exposure (−16% (95% CI: −25%, −5%)). Associations between self-reported activities and hourly PM_2.5_ are presented in S-Fig. 6.

## 4 Discussion

Our results indicate that wearable cameras can yield valid data that are associated with short-term variation in personal PM_2.5_ exposure. Our analyses resulted in two main findings. First, the high time resolution of the wearable camera data was particularly useful in identifying predictors of peak exposures. Wearable camera data identified a diverse range of activities and locations that could be difficult to capture using other single sources of information (e.g. questionnaire or GPS or temperature loggers used as stove use monitors (Ruiz-Mercado et al., 2012)). Second, men had higher exposures for most visual concepts, including those related to cooking and presence in the kitchen. However, the influence of cooking on exposures in men was not evident from the self-reported time activity data.

The vast majority of the household air pollution literature has focused on the influence of cooking activities on exposure for the primary cook (usually female) and children who spend large amounts of time with the primary cook, providing little data on exposures for adult men (Carter et al., 2017; Pope et al., 2017). Our results suggest that cooking-related activities contributed to high short-term exposures for men. Although men were not actively cooking and therefore did not report this activity in the questionnaire, they nonetheless spent time in microenvironments where cooking was taking place. The higher measured PM_2.5_ for men compared to women according to visual concepts related to food preparation and cooking may be explained by men limiting their time in these microenvironments to when cooking was taking place (e.g. immediately before mealtime), whereas women also spent time in these microenvironments (e.g. kitchen) when cooking was not occurring.

Mobility patterns and activities are notably different between men and women in this population (Sanchez et al., 2017). In a prior work based on GPS tracking in the same study population, we reported that men typically spend less time in the home, make more trips at high speed (e.g., in vehicle rather than walking), and travel further distances from the home compared to women (Sanchez et al., 2017). Nonetheless, our results here did not identify travel microenvironments as contributing to high short-term PM_2.5_ exposure for either sex. Other studies have identified commuting as contributing to high microenvironmental exposures for PM_2.5_, as well as for black carbon and ultrafine particles (Bekö et al., 2015; Nieuwenhuijsen et al., 2015; Pant et al., 2017). This difference may reflect the peri-urban setting of our study, which does not typically have high traffic congestion and emissions that would be encountered in urban environments. In comparison to previous studies focused on occupationally exposed populations (Semple et al., 2008), our data shed light on the relative contribution of PM_2.5_ in occupational settings in a sample of the general population in India. Five-minute average PM_2.5_ was positively associated with visual concepts related to presence in industry (for men) and informal work (for women), indicating the influence of occupational exposures. However, the strength of the associations for occupation-related visual concepts was weaker than for those related to cooking.

Beyond high temporal resolution, another advantage of wearable camera derived activity data is that the data collection does not depend on the questions asked in the field. Potentially, the data could (if participants consent) be re-used for other studies with different annotation protocols. This technology may have particular value for low-literacy populations, where feasibility is limited for participants to keep a written diary of activities. One disadvantage of the wearable camera technology is scalability due to the time-intensive process of manual annotation. However, scalability could be improved with future developments in machine learning to support automatic annotation (Bolanos et al., 2017). At the time of our study, we did not identify an annotation algorithm with sufficiently good performance for our study context, making manual annotation unavoidable. A further disadvantage of the wearable camera technology is potential discomfort and loss of privacy. Important ethical issues associated with using wearable cameras in research include informed consent (particularly with third parties), anonymity and confidentiality, data protection and privacy. These issues have been previously examined in detail (Kelly et al., 2013; Mok et al., 2015). Suggestions to address these issues include 1) limiting the scale and scope of data collection as much as possible; and 2) introducing firewalls around data to isolate them within the academic research context and to prevent participants from accessing and sharing their data (Kelly et al., 2013; Mok et al., 2015). We have taken both steps in our study.

Our study is based on two high temporally-resolved sources of information, the wearable camera and a continuous PM_2.5_ monitor, that are used in combination for the first time. A further strength of our study is the repeated measures design. The quality of our wearable camera-derived microenvironmental information was high; we used best practice for determining inter-rater agreement of photograph annotations via duplicate annotation by a distinct annotator and we had substantial to almost perfect agreement (S-Table 3) (Landis and Koch, 1977). We also conducted extensive post processing and quality control of the MicroPEM PM_2.5_ data (Salmon, 2017a). Our population is a sample of the general population in peri-urban South India, with likely good generalizability to similar populations (S-Table 2 shows study population in comparison to random sample of APCAPS cohort). Our results provide insights into predictors of exposure in multiple exposure settings, unlike studies focusing only on occupational or home environments or commuting.

Several methodological limitations should be considered while interpreting our results. The Autographer data was limited by the battery life of the device and therefore did not provide microenvironmental data for the full 24 h. The devices also provide low quality images in indoor conditions with low lighting; as a result, some visual concepts were potentially missed: 19% of images were uncodeable because of obstruction of the lens or low lighting conditions, which is consistent with other wearable camera studies (Doherty et al., 2013b). The regression models (Fig. 3) could not be adjusted by ambient PM_2.5_ because of collinearity with some activities and visual concepts with strong seasonal patterns (e.g., agricultural work). However, relative ranking of the influence of visual concepts on exposure is unlikely to be changed by adjustment with ambient values. We cannot rule out the possibility that participants changed their behavior as a result of wearing the camera. However, there was no indication of a trend in the number of photographs collected during each session, which might be expected if participants became more or less comfortable with wearing the device (S-Table 7). Similarly, differences in self-reported activities between people who participated in the panel study versus a random sample of the APCAPS cohort were modest (S-Table 8). Where there were statistically significant differences, they suggested that the panel study participants were slightly more active than the average for the APCAPS cohort. Female panel participants spent more time (~30 min) cooking, while male panel participants spent more time in the workplace (~108 min) compared to a sample of the APCAPS population.

In conclusion, our results indicate that microenvironmental data derived from wearable cameras can be valuable for understanding locations and activities that influence personal PM_2.5_ exposure. In particular, the high temporal resolution of these data is well suited for identifying microenvironments contributing to relatively short-term peak exposures. The contribution of food preparation and cooking to personal exposure among men in settings where biomass cooking fuel is common warrants further investigation. The use of wearable cameras is part of a promising movement towards the collection of densely sampled objective data with application for exposure science that may provide insights not possible with self-reported data.

## Appendix A Supplementary data

Supplementary material Image 1
