# Supplementary material for: Wearable camera-derived microenvironments in relation to personal exposure to PM2.5
Source: Environ Int. 2018 Aug;117:300–7. doi: 10.1016/j.envint.2018.05.021 (PMC6024072; doi:10.1016/j.envint.2018.05.021)

**Supporting Information**

Wearable camera-derived microenvironments in relation to personal exposure to PM_2.5_

Maëlle Salmon, ^1^ Carles Milà, ^1^ Santhi Bhogadi,^2^ Srivalli Addanki, ^2^ Pavitra Madhira, ^2^ Niharika Muddepaka, ^2^ Amaravathi Mora, ^2^ Margaux Sanchez,^1^ Sanjay Kinra,^4^ V. Sreekanth,^3^ Aiden Doherty,^5^ Julian D. Marshall, ^3^ Cathryn Tonne^1*^

1. ISGlobal, Universitat Pompeu Fabra, CIBER Epidemiología y Salud Pública, Barcelona, Spain.
2. Public Health Foundation of India, New Delhi, India
3. Department of Civil and Environmental Engineering, University of Washington, Seattle, WA, USA
4. Department of Non-communicable Disease Epidemiology, London School of Hygiene and Tropical Medicine, London, UK
5. Big Data Institute, Nuffield Department of Population Health, University of Oxford, Oxford, U.K.

## * Corresponding author: [Cathryn.tonne@isglobal.org](mailto:Cathryn.tonne@isglobal.org)


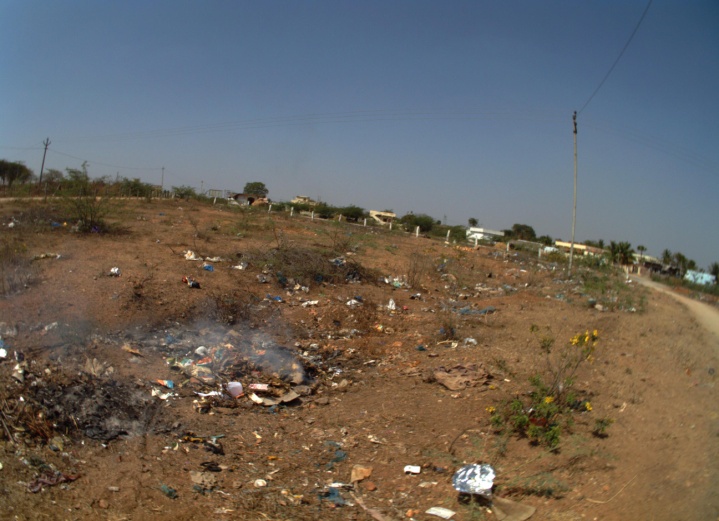

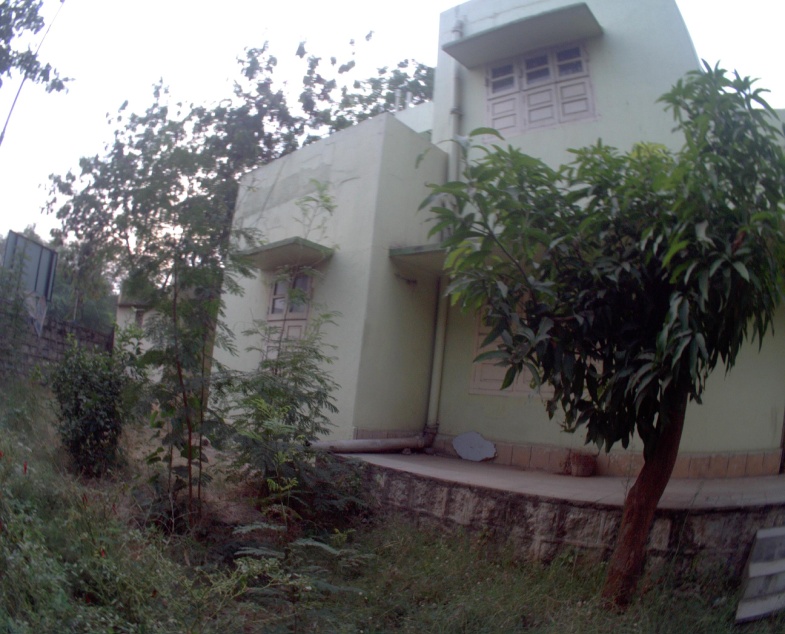

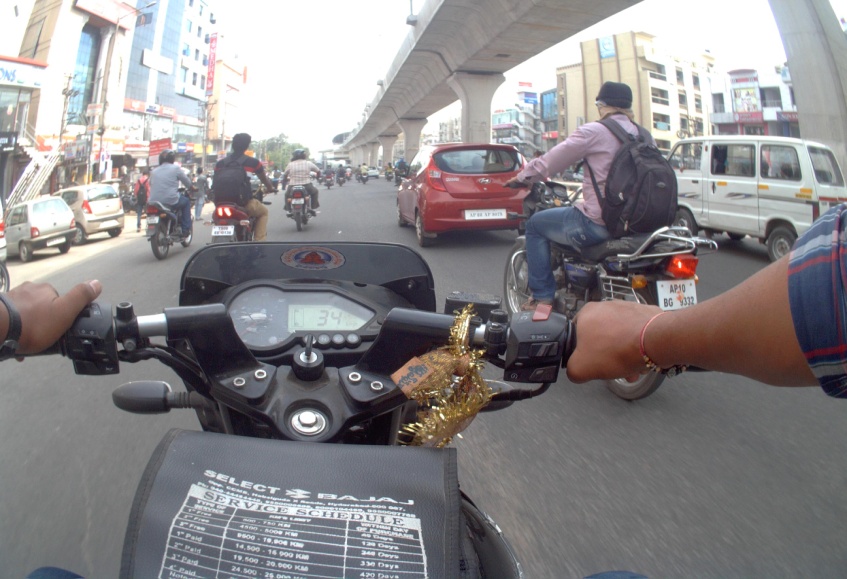

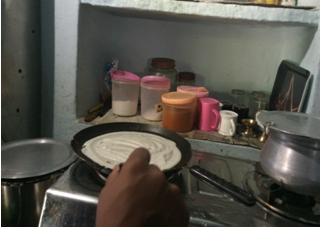


**S-Figure 1. Examples of photographs from wearable cameras**


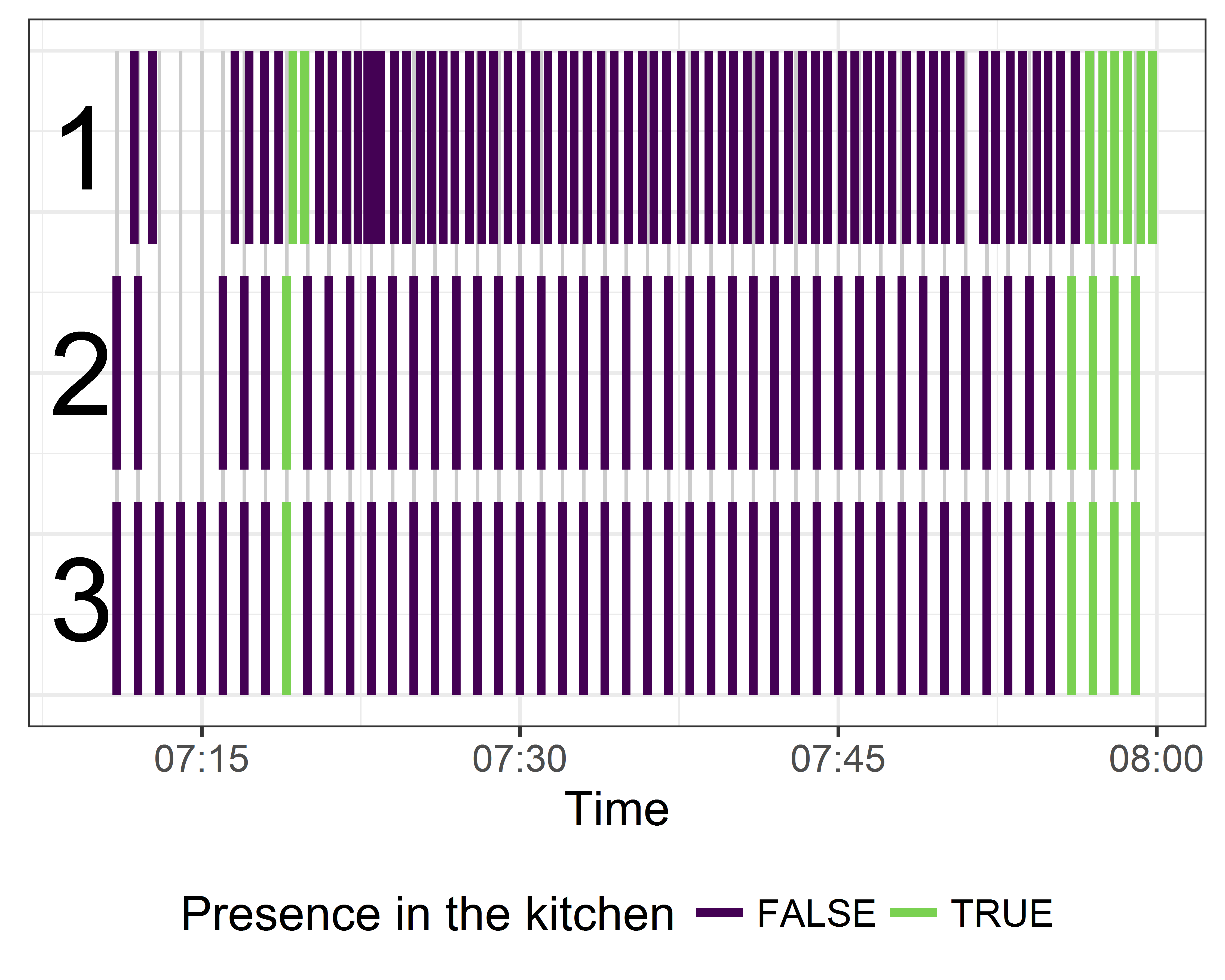


**S-Figure 2. Aggregation of wearable camera derived annotations. (1) Raw data after annotation, one value per photograph. (2) Data after aggregation to the minute, one value per minute, TRUE if any photograph had a TRUE value during that minute. (3) Data after filling of missing values with data from the latest 5 minutes before the minute.**

Extended methods: After annotation we had a raw dataset of annotation Boolean values by photograph (**S-Figure 2.1**). At that stage an annotation=TRUE means a photograph had a given annotation. We aggregated this dataset to first get annotation Boolean values by minute (here an annotation=TRUE means the annotation is assumed to have been present at some point during that minute) and then annotation average values by 5 minute intervals (0 meaning an annotation was absent in all 5 minutes, 1 that it was present in all of them). This final data was used in the regressions while the raw data was used for computing inter rater agreement. The 1 minute data was used to represent data coverage.

The aggregation from raw to 1 minute data happened with the following methods and assumptions. In the first step, for each minute, an annotation Boolean value, e.g. the value for the visual concept "Presence in the kitchen" was set to TRUE if any photograph within that minute included the annotation. This is the transition from 1 to 2 in the figure. In the second step (**S Figure 2.2 to 2.3**), minutes with no photographs (e.g. uncodable images) were imputed with the annotations of the last minute with photographs, up to 5 minutes earlier.

**
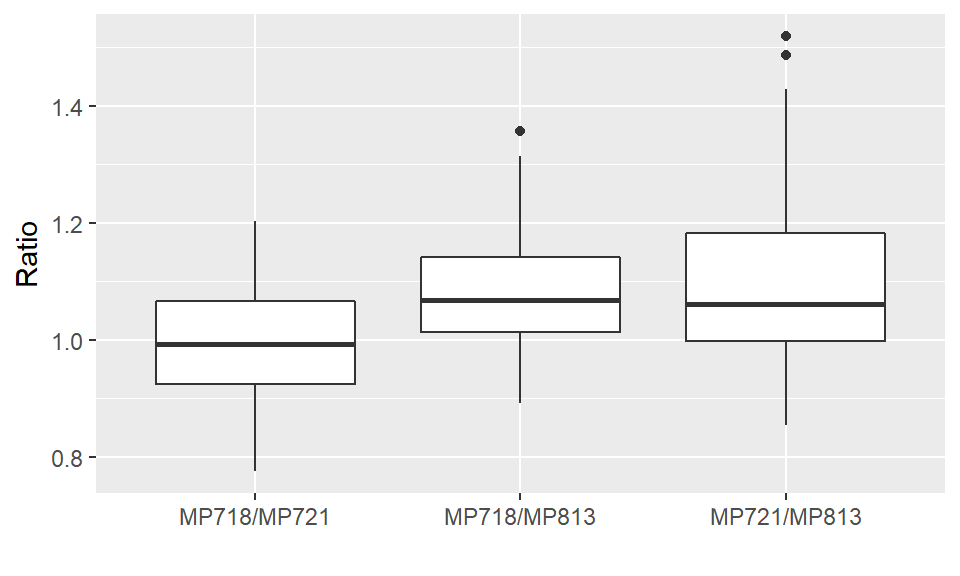
**

**S-Figure 3. Boxplots of ratios derived from 10 s time series among three collocated MicroPEM devices**

**S-Table 1. Summary statistics from three collocated MicroPEM devices (µg/m^3^)**

| Device | Mean | Median | SD | CV* | Min | Max | Range | Q25 | Q75 | IQR |
| --- | --- | --- | --- | --- | --- | --- | --- | --- | --- | --- |
| MP721 | 57.7 | 48.1 | 24.8 | 0.4 | 37.5 | 156.5 | 119.1 | 43.6 | 55.8 | 12.2 |
| MP718 | 56.0 | 49.3 | 19.1 | 0.3 | 39.7 | 123.8 | 84.1 | 44.7 | 55.9 | 11.2 |
| MP813 | 51.2 | 46.4 | 14.5 | 0.3 | 36.8 | 103.0 | 66.2 | 42.1 | 52.7 | 10.6 |

*CV: coefficient of variation (unitless); Q25: 25^th^ percentile; Q75: 75^th^ percentile

**
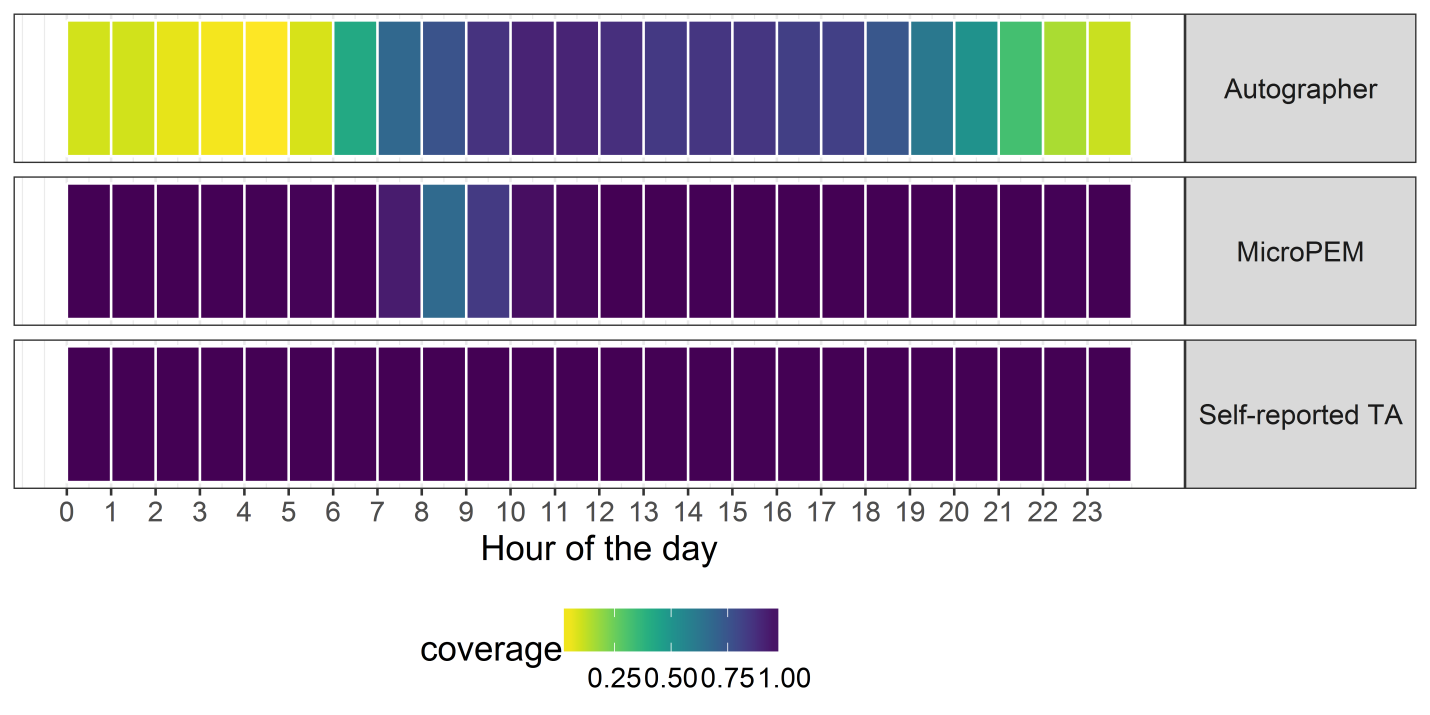
**

**S-Figure 4. Time coverage of the wearable camera, personal PM_2.5_ monitor, and self-reported time activity.**

**S-Table 2. Characteristics of panel study participants, those excluded from the panel, and a random sample of the APCAPS cohort**

|  | Women |  |  | Men |  |  | All |  |  |  |
| --- | --- | --- | --- | --- | --- | --- | --- | --- | --- | --- |
|  | Panel | Excluded | APCAPS^1^ | Panel | Excluded | APCAPS^1^ | Panel | Excluded | APCAPS^1^ | P-value^2^ |
| N | 23 | 8 | 215 | 22 | 7 | 323 | 45 | 15 | 538 |  |
| Age (years), m (sd) | 48 (9) | 48 (13) | 44 (12) | 40 (17) | 28 (11) | 38 (17) | 44 (14) | 39 (16) | 40 (15) | 0.12 |
| Marital status, married, n (%) | 17 (74) | 6 (75) | 148 (69) | 14 (64) | 1 (14) | 189 (59) | 31 (69) | 7 (47) | 337 (63) | 0.43 |
| Education level, illiterate, n (%) | 18 (78) | 6 (75) | 162 (75) | 6 (27) | 0 (0) | 89 (28) | 24 (53) | 6 (40) | 251 (47) | 0.44 |
| Current smoker, n (%) | 0 (0) | 0 (0) | 0 (0) | 6 (27) | 0 (0) | 81 (25) | 6 (13) | 0 (0) | 81 (15) | 1 |
| Primary occupation, n (%) |  |  |  |  |  |  |  |  |  | 0.26 |
| Unemployed | 2 (9) | 2 (25) | 51 (24) | 2 (9) | 2 (29) | 59 (18) | 4 (9) | 4 (27) | 110 (20) |  |
| Unskilled manual | 16 (70) | 4 (50) | 124 (58) | 8 (36) | 1 (14) | 100 (31) | 24 (53) | 5 (33) | 224 (42) |  |
| Semi-skilled manual | 3 (13) | 1 (12) | 29 (13) | 3 (14) | 3 (43) | 60 (19) | 6 (13) | 4 (27) | 89 (17) |  |
| Skilled manual | 1 (4) | 1 (12) | 6 (3) | 8 (36) | 1 (14) | 76 (24) | 9 (20) | 2 (13) | 82 (15) |  |
| Non manual | 1 (4) | 0 (0) | 5 (2) | 1 (5) | 0 (0) | 28 (9) | 2 (4) | 0 (0) | 33 (6) |  |
| Agriculture-related occupation, n (%) | 16 (70) | 4 (50) | 108 (50) | 6 (27) | 0 (0) | 109 (34) | 22 (49) | 4 (27) | 217 (40) | 0.27 |
| Body mass index (kg/m2), n (%) |  |  |  |  |  |  |  |  |  | 0.76 |
| <18.5 | 6 (26) | 1 (12) | 63 (29) | 6 (27) | 3 (43) | 110 (34) | 12 (27) | 4 (27) | 173 (32) |  |
| 18.5–23.0 | 9 (39) | 4 (50) | 89 (41) | 12 (55) | 4 (57) | 145 (45) | 21 (47) | 8 (53) | 234 (43) |  |
| >=23.0 | 8 (35) | 3 (38) | 58 (27) | 3 (14) | 0 (0) | 64 (20) | 11 (24) | 3 (20) | 122 (23) |  |
| Primary fuel use, n (%) ^3^ |  |  |  |  |  |  |  |  |  |  |
| Biomass | 3 (13) | 4 (50) | 92 (43) | 12 (55) | 4 (57) | 159 (49) | 15 (33) | 8 (53) | 251 (47) | 0.09 |
| LPG | 22 (96) | 7 (88) | 188 (87) | 17 (77) | 5 (71) | 267 (83) | 39 (87) | 12 (80) | 455 (85) | 0.83 |
| Others | 5 (22) | 1 (12) | 35 (16) | 4 (18) | 2 (29) | 51 (16) | 9 (20) | 3 (20) | 86 (16) | 0.53 |

^1^ CHAI participants are a random sample of the APCAPS cohort.

^2^ Test for differences between participants included in the panel and CHAI. P-value of Fisher exact test for all variables except age (t-test). Primary fuel use types have been tested separately because more than one fuel type is possible.

^3^ More than one primary fuel type is possible.

**S-Table 3. Inter-rater agreement (Kappa) by visual concept**

| code | category | no. of photographs | agreement prop. | Kappa | p-value |
| --- | --- | --- | --- | --- | --- |
| biomass cooking unit | CK | 1172 | 1.00 | 0.94 | <0.001 |
| LPG stove | CK | 2143 | 1.00 | 0.91 | <0.001 |
| other cooking unit | CK | 927 | 1.00 | 0.92 | <0.001 |
| food preparation | CK | 3459 | 0.99 | 0.89 | <0.001 |
| eating | CK | 5560 | 0.99 | 0.93 | <0.001 |
| presence in the kitchen | CK | 8893 | 0.99 | 0.96 | <0.001 |
| presence at office or shop | OP | 3345 | 1.00 | 0.98 | <0.001 |
| presence at work field | OP | 6522 | 1.00 | 0.96 | <0.001 |
| presence in industry | OP | 10554 | 1.00 | 0.99 | <0.001 |
| presence in informal work | OP | 4579 | 0.99 | 0.93 | <0.001 |
| diesel generator | PM | 6 | 1.00 | 0.67 | <0.001 |
| smoking | PM | 552 | 1.00 | 0.81 | <0.001 |
| visible flame or smoke | PM | 611 | 1.00 | 0.83 | <0.001 |
| travel by bus | TP | 0 | 1.00 | NaN | NaN |
| travel by bicycle | TP | 777 | 1.00 | 0.92 | <0.001 |
| travel by auto-rickshaw | TP | 1524 | 0.99 | 0.61 | <0.001 |
| travel by motorcycle | TP | 1488 | 1.00 | 0.92 | <0.001 |
| participant presence on road | TP | 15957 | 0.99 | 0.94 | <0.001 |
| NA | IO | 0 | 0.95 | 0.92 | <0.001 |

##### Categories are travel (TP), occupation (OP), cooking (CK), indoor/outdoor location (IO) and presence of other combustion (PM). Kappa not calculated for IO, for which there was a single Kappa value for non codable/indoors/outdoors/in vehicle/mixed together since these concepts are mutually exclusive.

**S-Table 4. Time spent per day by visual concept according to sex**

|  | Women: Total no. of minutes | | Prop. of minutes (%) | | Men: Total no. of minutes | | Prop. of minutes (%), | |
| --- | --- | --- | --- | --- | --- | --- | --- | --- |
| Visual concept | median | sd | median | sd | median | sd | median | sd |
| **Cooking** |  |  |  |  |  |  |  |  |
| Biomass cooking unit | 0 | 27 | 0 | 4 | 2 | 19 | 0 | 2 |
| LPG stove | 20 | 32 | 3 | 5 | 3 | 24 | 0 | 5 |
| Other cooking unit | 0 | 34 | 0 | 5 | 0 | 8 | 0 | 2 |
| Food preparation | 48 | 57 | 7 | 9 | 7 | 18 | 1 | 2 |
| Eating | 40 | 24 | 6 | 3 | 34 | 26 | 6 | 4 |
| Presence in the kitchen | 77 | 149 | 11 | 21 | 7 | 52 | 1 | 13 |
| **Travel** |  |  |  |  |  |  |  |  |
| Travel by bus | 0 | 0 | 0 | 0 | 0 | 10 | 0 | 1 |
| Travel by bicycle | 0 | 1 | 0 | 0 | 0 | 28 | 0 | 4 |
| Travel by auto rickshaw | 0 | 5 | 0 | 1 | 0 | 48 | 0 | 6 |
| Travel by motorcycle | 0 | 7 | 0 | 1 | 0 | 37 | 0 | 6 |
| Travel by car | 0 | 0 | 0 | 0 | 0 | 16 | 0 | 3 |
| Presence on road | 55 | 113 | 8 | 16 | 85 | 116 | 14 | 16 |
| **Occupation** |  |  |  |  |  |  |  |  |
| Presence at office or shop | 0 | 36 | 0 | 5 | 0 | 141 | 0 | 22 |
| Presence at work field | 0 | 163 | 0 | 25 | 0 | 108 | 0 | 15 |
| Presence in industry | 0 | 7 | 0 | 3 | 0 | 198 | 0 | 28 |
| Presence in informal work | 0 | 34 | 0 | 5 | 0 | 131 | 0 | 18 |
| **Presence of non-cooking combustion** | | |  |  |  |  |  |  |
| Diesel generator | 0 | 0 | 0 | 0 | 0 | 1 | 0 | 0 |
| Smoking | 0 | 5 | 0 | 1 | 0 | 24 | 0 | 4 |
| Visible flame or smoke | 0 | 8 | 0 | 1 | 0 | 23 | 0 | 4 |
| **Location** |  |  |  |  |  |  |  |  |
| Indoors | 436 | 230 | 64 | 28 | 292 | 229 | 49 | 28 |
| Outdoors | 210 | 203 | 32 | 28 | 240 | 204 | 42 | 28 |
| In vehicle | 0 | 5 | 0 | 1 | 0 | 75 | 0 | 10 |
| Mixed | 2 | 21 | 0 | 3 | 1 | 10 | 0 | 1 |

**S-Table 5. Time spent per day by self-reported activity according to sex**

|  | Women: Total median (h) | sd | Prop. of minutes (%), median | sd | Men: Total median (h) | sd | Prop. of minutes (%), median | sd |
| --- | --- | --- | --- | --- | --- | --- | --- | --- |
| **Activities** |  |  |  |  |  |  |  |  |
| Cooking | 2 | 1.2 | 8.3 | 4.8 | 0 | 0.2 | 0.0 | 1.0 |
| Household chores | 3 | 2.3 | 12.5 | 9.5 | 0 | 1.2 | 0.0 | 4.9 |
| Work | 0 | 3.1 | 0.0 | 12.9 | 6 | 4.5 | 25.0 | 18.8 |
| Study | 0 | 0.0 | 0.0 | 0.0 | 0 | 0.2 | 0.0 | 0.9 |
| Playing | 0 | 0.1 | 0.0 | 0.4 | 0 | 0.0 | 0.0 | 0.0 |
| Travel | 0 | 0.4 | 0.0 | 1.5 | 1 | 1.9 | 4.2 | 7.8 |
| Sedentary | 7 | 3.5 | 29.2 | 14.8 | 7 | 4.2 | 29.2 | 17.4 |
| Sleep | 9 | 1.4 | 37.5 | 5.7 | 9 | 1.6 | 37.5 | 6.6 |
| Personal care | 1 | 0.9 | 4.2 | 3.6 | 1 | 1.0 | 4.2 | 4.0 |
| Walking | 0 | 1.4 | 0.0 | 5.7 | 1 | 2.1 | 4.2 | 8.6 |
| Other | 0 | 0.6 | 0.0 | 2.5 | 0 | 1.4 | 0.0 | 5.7 |
| **Locations** |  |  |  |  |  |  |  |  |
| Indoor - home | 19 | 4.4 | 79.2 | 18.2 | 14 | 4.6 | 58.3 | 19.0 |
| Indoor - classroom/office/shop | 0 | 1.1 | 0.0 | 4.6 | 0 | 2.5 | 0.0 | 10.6 |
| Playground/complex/compound | 2 | 3.6 | 8.3 | 14.8 | 0 | 2.9 | 0.0 | 12.2 |
| Outdoor in village | 1 | 1.9 | 4.2 | 7.7 | 2 | 3.2 | 8.3 | 13.3 |
| Outdoor in fields | 0 | 2.6 | 0.0 | 10.9 | 0 | 3.3 | 0.0 | 13.7 |
| Workplace | 0 | 1.8 | 0.0 | 7.3 | 0 | 4.2 | 0.0 | 17.6 |
| Travel - closed windows | 0 | 0.0 | 0.0 | 0.0 | 0 | 0.4 | 0.0 | 1.6 |
| Travel - other | 0 | 0.4 | 0.0 | 1.5 | 0 | 1.7 | 0.0 | 7.1 |


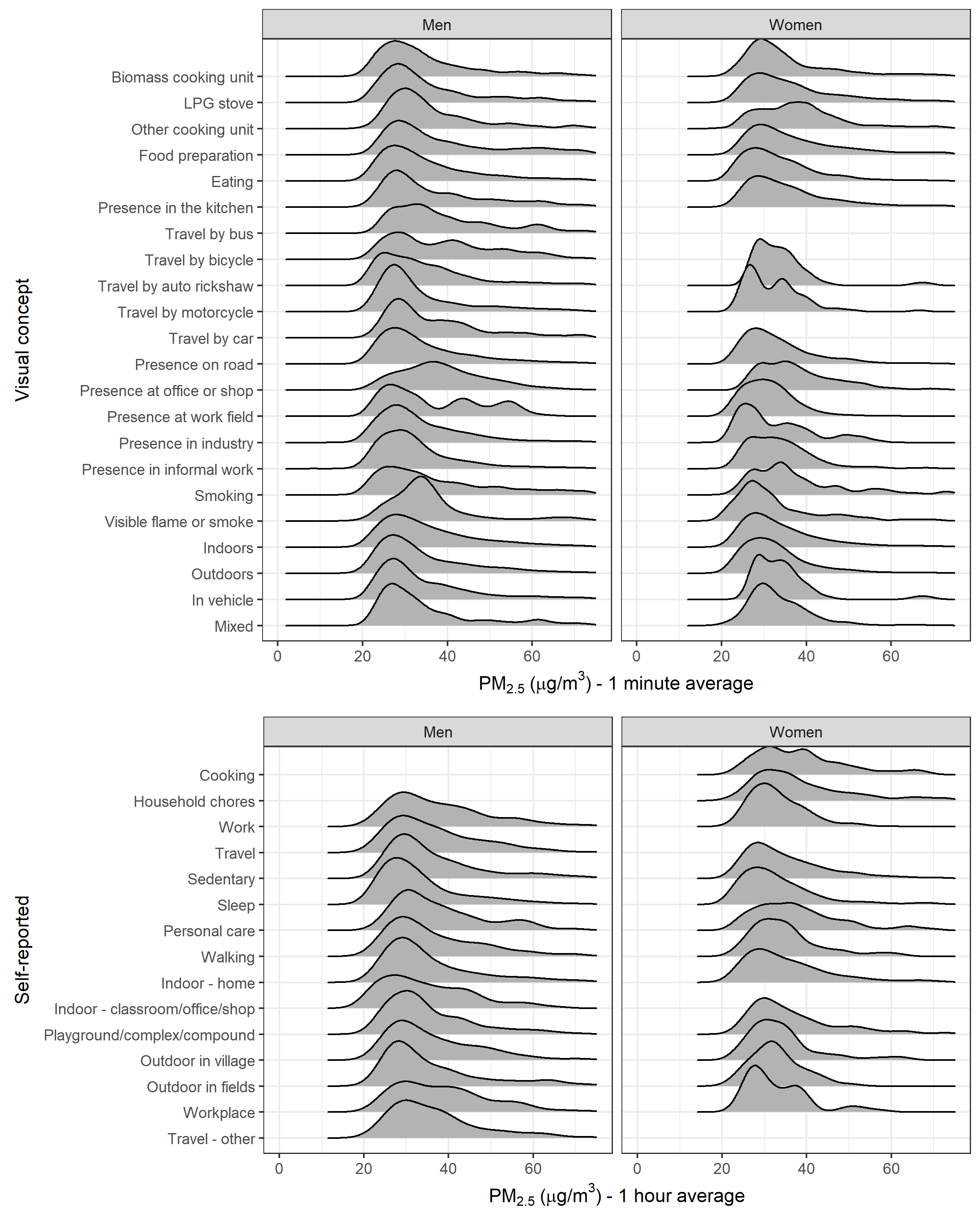
S-Figure 5. Distribution of PM_2.5_ by visual concept (A) and self-reported activities and locations (B). Figure truncated at 75 μg/m^3^ for visualization purposes. Visual concepts and activities with an occurrence <50 are excluded.

**S-Table 6. Measured PM_2.5_ exposure (μg/m^3^) according to self-reported activity and location**

|  | Women: Mean (sd) | Median | No. of hours | Men: Mean (sd) | Median | No. of hours |
| --- | --- | --- | --- | --- | --- | --- |
| **Activities** |  |  |  |  |  |  |
| Cooking | 54 (53) | 39 | 183 | 43 (15) | 15 | 4 |
| Household chores | 49 (43) | 37 | 342 | 49 (68) | 68 | 39 |
| Work | 34 (9) | 32 | 237 | 46 (42) | 42 | 527 |
| Study | NA | NA | NA | 32 (6) | 6 | 3 |
| Playing | 35 (NA) | 35 | 1 | NA | NA | NA |
| Travel | 34 (4) | 34 | 9 | 40 (16) | 16 | 152 |
| Sedentary | 39 (25) | 33 | 824 | 46 (47) | 47 | 750 |
| Sleep | 36 (21) | 31 | 533 | 37 (26) | 26 | 562 |
| Personal care | 49 (37) | 38 | 83 | 44 (26) | 26 | 115 |
| Walking | 36 (13) | 34 | 116 | 49 (58) | 58 | 146 |
| Other | 37 (12) | 31 | 9 | 38 (17) | 17 | 32 |
| **Locations** |  |  |  |  |  |  |
| Indoor - home | 41 (33) | 34 | 1557 | 42 (33) | 33 | 1160 |
| Indoor - classroom/office/shop | 34 (8) | 33 | 25 | 36 (14) | 14 | 83 |
| Playground/complex/compound | 44 (31) | 35 | 294 | 55 (80) | 80 | 148 |
| Outdoor in village | 35 (11) | 33 | 148 | 42 (39) | 39 | 283 |
| Outdoor in fields | 34 (10) | 32 | 143 | 43 (36) | 36 | 179 |
| Workplace | 33 (8) | 31 | 68 | 50 (52) | 52 | 298 |
| Travel - closed windows | NA | NA | NA | 37 (10) | 10 | 8 |
| Travel – other | 34 (4) | 34 | 9 | 42 (20) | 20 | 118 |


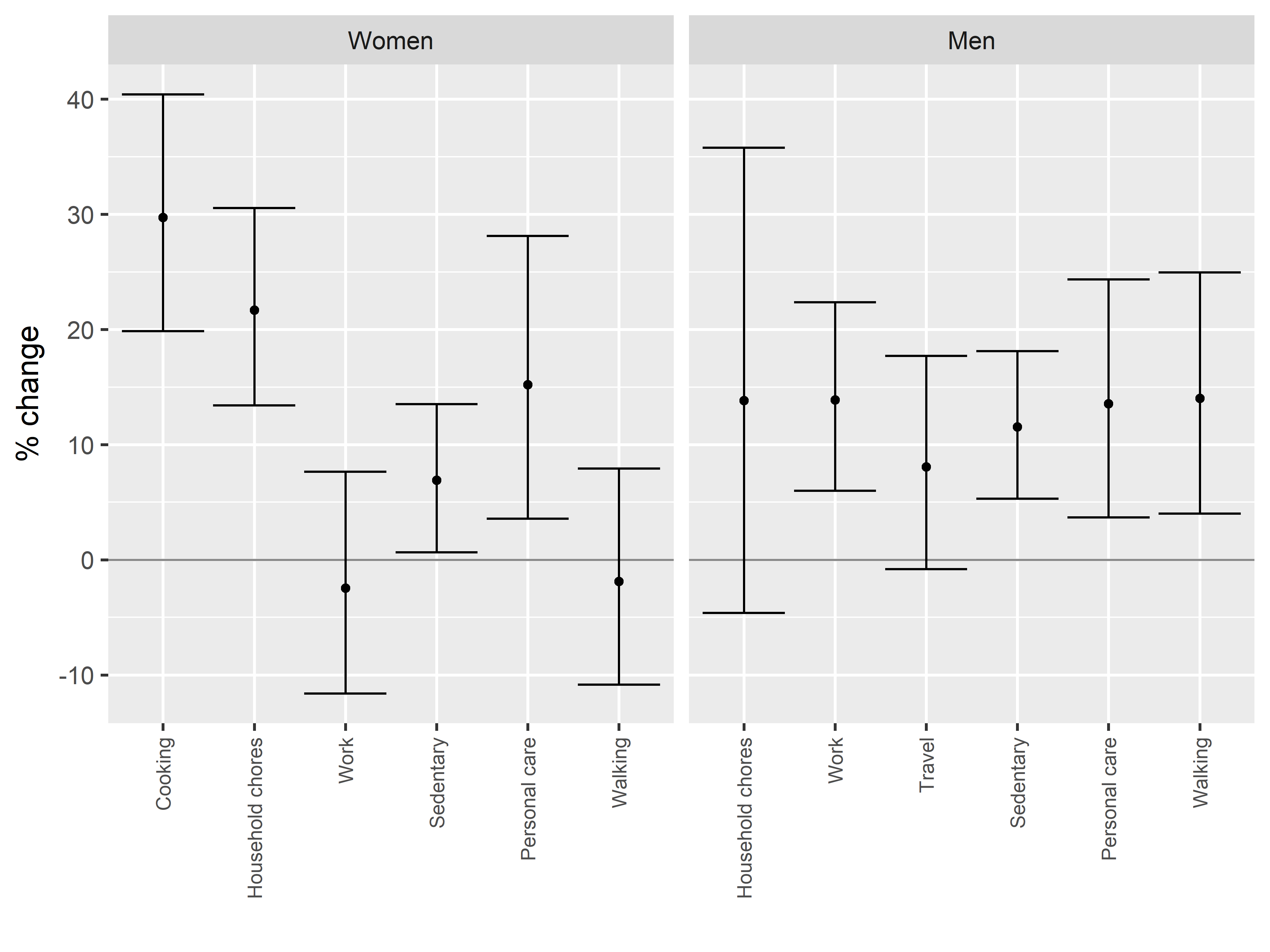


S-Figure 6. Percent change and 95% confidence intervals in hourly average PM_2.5_ associated with self-reported activities. Regression estimates are mutually adjusted.

S-Table 7. Number of photographs taken by monitoring session according to sex

| Session | Women:  Mean (sd) | Median | No. of participants | Men:  Mean (sd) | Median | No. of participants |
| --- | --- | --- | --- | --- | --- | --- |
| 1 | 1290 (452) | 1092 | 22 | 1280 (289) | 1262 | 22 |
| 2 | 1589 (447) | 1683 | 20 | 1482 (551) | 1393 | 20 |
| 3 | 1487 (540) | 1478 | 18 | 1355 (379) | 1309 | 16 |
| 4 | 1555 (439) | 1614 | 18 | 1371 (616) | 1204 | 13 |
| 5 | 1298 (581) | 1194 | 14 | 1177 (411) | 1144 | 14 |
| 6 | 1352 (361) | 1369 | 15 | 1276 (351) | 1310 | 15 |

**S-Table 8. Average time spent per day by self-reported activity compared to random sample of APCAPS cohort**

|  | Women:  Panel | APCAPS | P-value* | Men: Panel | APCAPS | P-value* |
| --- | --- | --- | --- | --- | --- | --- |
| Number of participants (N) | 23 | 141 |  | 22 | 212 |  |
| **Activities (mean (sd) hours)** |  |  |  |  |  |  |
| Cooking | 1.8 (0.8) | 1.3 (0.9) | 0.03 | 0.1 (0.3) | 0.1 (0.6) | 0.46 |
| Household chores | 3.2 (1.9) | 2.6 (1.9) | 0.15 | 0.4 (0.9) | 0.2 (0.7) | 0.07 |
| Work | 2.5 (2.9) | 3 (3.2) | 0.6 | 5.6 (3.9) | 4.2 (3.7) | 0.06 |
| Study | 0 (0) | 0.1 (0.4) | 0.49 | 0 (0) | 0.1 (0.8) | 0.28 |
| Playing | 0 (0) | 0 (0.1) | 0.55 | 0 (0) | 0 (0.2) | 0.43 |
| Travel | 0.1 (0.2) | 0.3 (0.7) | 0.5 | 1.5 (1.2) | 1.2 (1.4) | 0.16 |
| Sedentary | 7.5 (2.7) | 7.6 (3) | 0.7 | 6.8 (3.8) | 8.4 (3.5) | 0.06 |
| Sleep | 8.8 (0.8) | 8.8 (1.2) | 0.65 | 9.1 (1) | 8.7 (1.3) | 0.32 |
| Personal care | 0.8 (0.7) | 0.9 (0.8) | 0.42 | 1.1 (0.6) | 1 (0.7) | 0.77 |
| Walking | 1.1 (1.1) | 1.1 (1.2) | 0.9 | 1.3 (1) | 1.6 (1.8) | 0.91 |
| Other | 0 (0.2) | 0.1 (0.3) | 0.71 | 0.3 (0.5) | 0.1 (0.4) | <0.01 |
| **Locations (mean (sd) hours)** |  |  |  |  |  |  |
| Indoor - home | 17.8 (3.7) | 17.2 (3.7) | 0.34 | 14.2 (3.2) | 14.9 (3.8) | 0.21 |
| Indoor - classroom/office/shop | 0.2 (0.7) | 0.3 (1.4) | 0.51 | 0.8 (2.2) | 0.8 (2.3) | 0.29 |
| Playground/complex/compound | 3.1 (3.1) | 2.9 (2.5) | 0.9 | 1.6 (2) | 1.8 (2.1) | 0.67 |
| Outdoor in village | 1.7 (1.8) | 2 (2.1) | 0.73 | 2.5 (1.9) | 3 (2.6) | 0.61 |
| Outdoor in fields | 1.1 (1.8) | 1.4 (2.2) | 0.98 | 1.9 (2.4) | 2.1 (3.1) | 0.57 |
| Workplace | 0.7 (1.2) | 1 (2.1) | 0.33 | 3.3 (3.5) | 1.5 (2.7) | 0.01 |
| Travel - closed windows | 0 (0) | 0 (0.4) | 0.36 | 0.1 (0.2) | 0.1 (0.4) | 0.34 |
| Travel - other | 0.1 (0.2) | 0.3 (0.6) | 0.72 | 1.2 (1) | 1.1 (1.6) | 0.12 |

* P-value corresponds to a Mann-Whitney test between the self-reported times in the panel and the random subsample of the APCAPS cohort.

|  |  |  |  |  |  |  |
| --- | --- | --- | --- | --- | --- | --- |


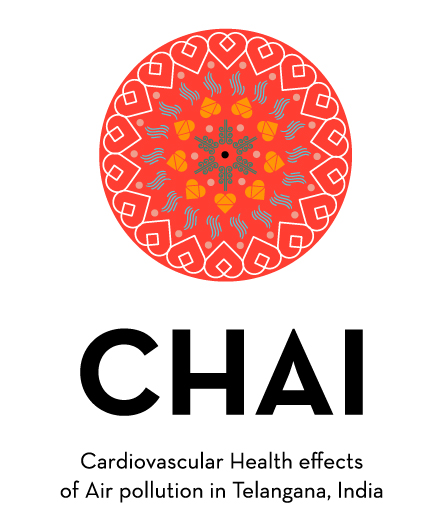


CHAI Project Autographer Coding Protocol

Developed by: Barcelona Institute for Global Health, Indian Institute for Public Health, Hyderabad

Research leading to this protocol received funding from the European Research Council under ERC Grant Agreement number 336167 for the CHAI Project

TABLE OF CONTENTS

**1. Introduction**

**2. Ethics**

**2.1 Prescribed reading for all annotators**

**3. Training Overview**

**4. Resources Required**

**4.1 Computer Requirements**

**4.2 XnViewMP**

**4.3 Setting up Software**

**4.3.1 Format of annotation schema txt file**

**4.3.2 Importing the list of codes**

**4.3.3 Adding category information to metadata export**

**4.3.4 Changing the layout**

**5. Browser Training**

**5.1 Browser access**

**5.2 Adding annotations**

**5.3 Exporting the annotations**

**6. Protocol Training**

**6.1 General Guidelines**

**6.1.1 Coding Photos**

**6.1.2 Calibration Photos**

**6.1.3 General Codes**

**6.2 Passes**

**6.2.1 PASS 1: Cooking**

**6.2.2 PASS 2: Travel**

**6.2.3 PASS 3: Occupation**

**6.2.4 PASS 4: Presence of other combustion**

**6.2.5. Pass 5: Indoor/Outdoor Pass**

**7. List of Codes**

**8. Example Images**

# 1.Introduction

Autographer is a wearable wide angle lens camera that takes photos at fixed intervals or in response to a change in the wearer’s environment. Autographer images may provide detailed and objectively measured time activity data for purposes of research. In order to analyse these data, images must first be sorted and screened for activities of interest. The XnViewMP Browser has been designed for this purpose. The browser allows users to categorise images into albums that can be tagged with descriptive keywords. Annotated Autographer data can them be extracted from the browser’s database and used for analysis.

To ensure reliability and efficiency in the tagging process, a coding protocol has been developed by the CHAI study team at the Barcelona Institute for Global Health (ISGlobal) and the Indian Institute of Public Health (IIPH), Hyderabad, India. This protocol is adapted from a protocol developed by the Center for Wireless and Population Health Systems, University of California at San Diego. The protocol enables photos to be tagged based on the wearer’s physical location (indoor vs outdoor), surroundings, and activity.

Annotators using this protocol to annotate wearable camera data must first be trained and certified. The protocol includes information on annotator training, the certification process, system requirements, and related readings.

# 2.Ethics

Researchers using visual (photographic) data should be aware of the relevant ethical issues. It is beyond the scope of this protocol to present a detailed discussion of the ethical issues that arise when using visual data in research; however, central principles are mentioned that guide ethical research and which form the decision making of most research ethics boards

The three core principles involve a researcher’s responsibility to:

(1) respect people’s autonomy (the right of others to make their own informed decision);

(2) non-maleficence (not doing harm or avoiding personal risks)

(3) justice (ensuring that the benefits and burdens are equitable, including across those who get invited vs. not invited to participate).

These core principles are often operationalized as a series of professional guidelines:

1. researchers should strive to protect the rights, privacy, dignity and well-being of those that they study;
2. research should be based on voluntary informed consent
3. personal information should be treated confidentially and, wherever possible, participants should be anonymized unless they choose to be identified;
4. research participants should be informed of the extent to which anonymity and confidentiality can be assured in publication and dissemination and of the potential re-use of data.

Individuals involved in coding wearable camera data should complete a basic tutorial on research ethics. For example, a free online tutorial is available from the US NIH.

## 2.1 Required reading for all annotators

**NIH research ethics online course**

[Available at: <http://researchethics.od.nih.gov/ConfirmBrowse.aspx>, click on browse course to enter]

**An ethical framework for automated, wearable cameras in health behaviour research.**

Paul Kelly, Simon Marshall, Hannah Badland et al. Am J PRev Med 2013; 44(3):314-319

<http://dx.doi.org/10.1016/j.amepre.2012.11.006>

**Too much information: visual research ethics in the age of wearable cameras.**

Mok, T.M., Cornish, F., Tarr, J., 2015. Integr. Psychol. Behav. Sci. 49, 309–322,

http://dx.doi.org/10.1007/s12124-014-9289-8.

# 3. TRAINING OVERVIEW

Before Autographer annotators start this training, it is recommended they wear the Autographer for a few days to collect data on their own behaviors. These data can be used to practice coding during the training process.

The coding process involves learning to annotate the content of the photos applying different categories. The first step in the training is an orientation to XnViewMP Browser. This is achieved by completing the Guided Browser Training. After this initial introduction to the Browser, users will learn the Autographer Coding Protocol. Using this protocol, Users will annotate photos that they collected prior to the start of the training.

Once the users are comfortable with the Browser and Annotation Protocol, they will need to be certified as annotators. Users should score Kappa > 85% in each category compared to the main annotator before moving on to main study data. Those who score lower than 85% must be re-trained and re-tested.

This training process may take 25 to 30 hours. Users will need ample time to learn the coding protocol and practice coding training data before they can become certified.

# 4. RESOURCES REQUIRED

##

## 4.1 Computer Requirements

An Intel quad core processor with any Operating System (Windows, Mac OS X and Linux).

## 4.2 XnViewMP

XnViewMP is a freeware for non- commercial use. It can be downloaded at:

<http://www.xnview.com/en/xnviewmp/#downloads>

**4.3 Setting up the Software**

**4.3.1 Format of annotation schema txt file**

The Autographer Coding Protocol for CHAI project categories is included in this protocol. If you need to develop your own categories we suggest you follow the steps outlined in this protocol ie. define the behaviors or environments, come up with examples of the behaviors/environments and develop clear identification rules, indicate exceptions to the rules, test whether the rules lead to reliable coding, re-assess rules if necessary and re-test. The first step is to prepare the annotation schema txt file with all categories and subcategories of interest.

Format of annotation schema file should be as follows

Main category1

Sub category1.1

Sub category1.2

Sub category1.3

Main category2

Subcategory2.1

Subcategory2.2

Subcategory2.3

For example we have travel as main category and travel by bus, travel by bicycle, and travel by auto are the subcategories under travel. Similarly, Occupation is a main category and presence at office or shop and presence at work field are subcategories under occupation; the categories txt file should look like

Travel

Travel by bus

Travel by bicycle

Travel by auto

Occupation

Presence at Office or Shop

Presence at Work Field

Once done with all categories save it as a text file.

**4.3.2 Importing the list of codes**

To be done in the “categories” pane.


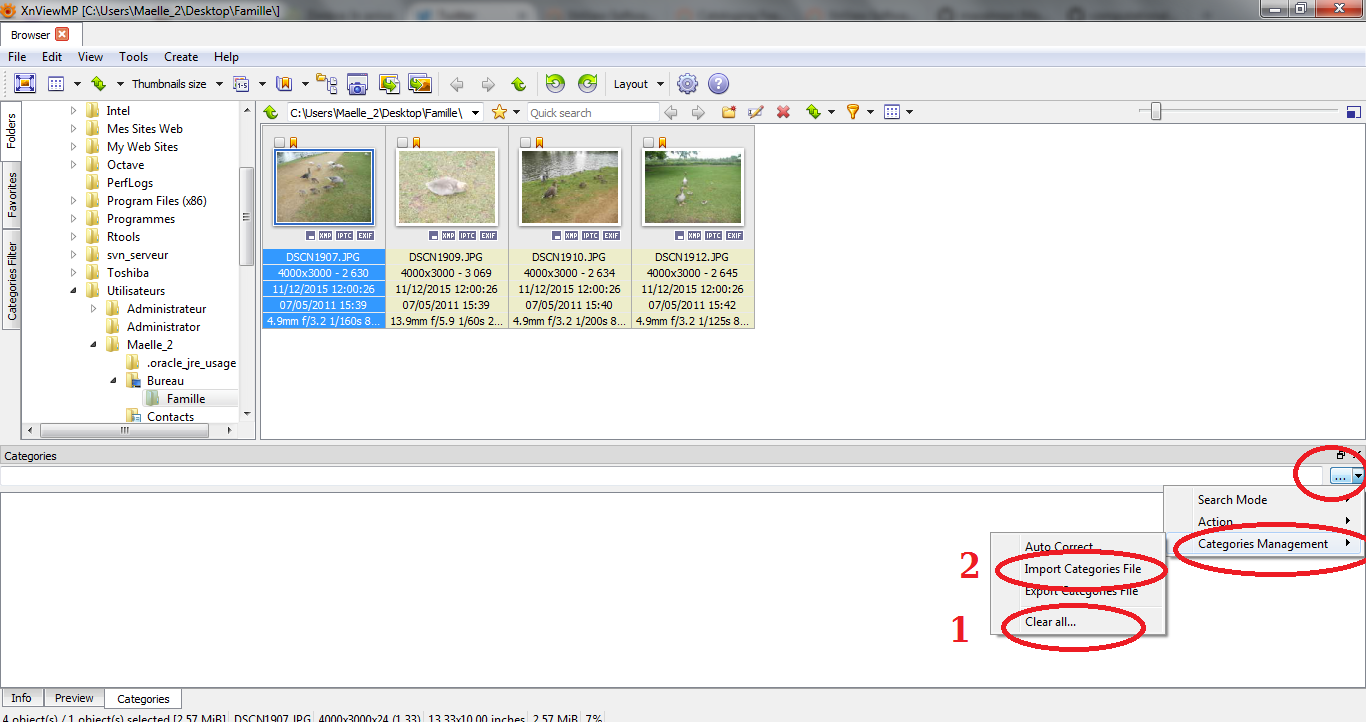


Click on the arrow, then on “Categories Management” and

1. Clear all so that all existing categories are erased.
2. Import categories file which has all relevant codes.

You should now see the categories with codes.
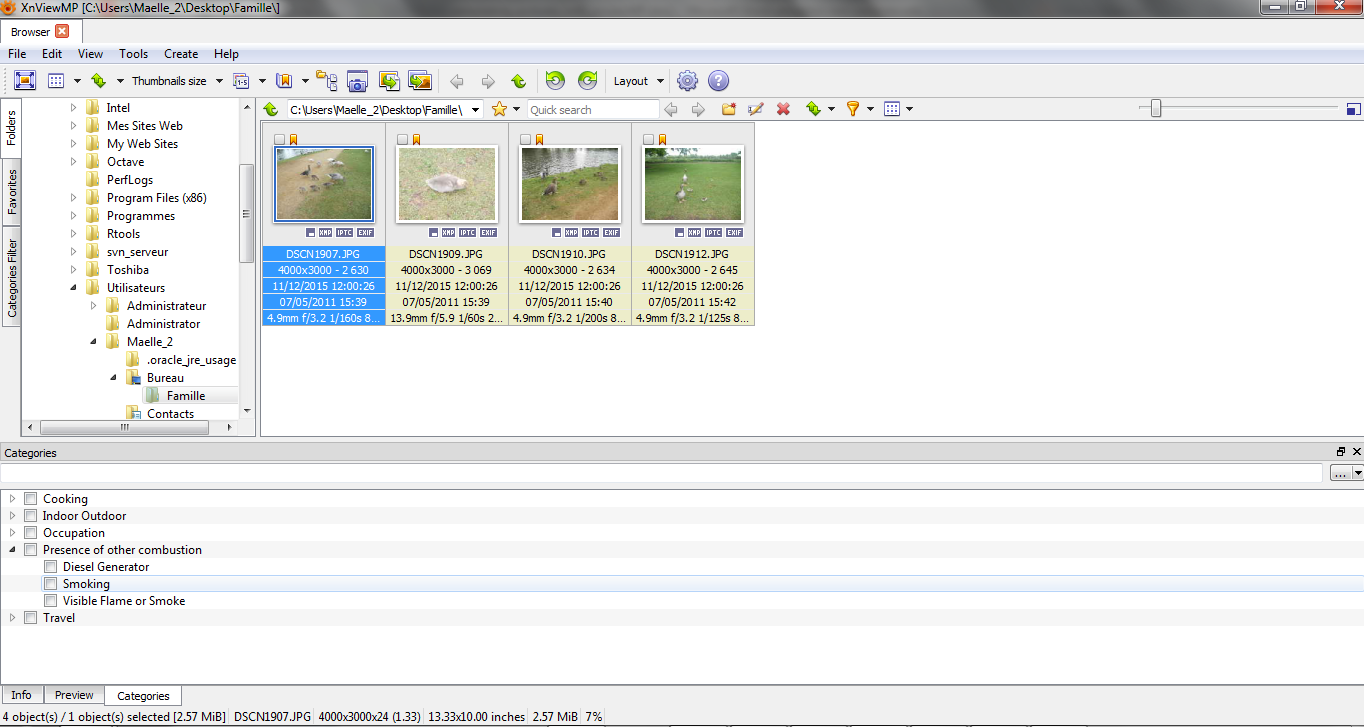


## 4.3.3 Adding category information to metadata export

Go to Tools/Settings.


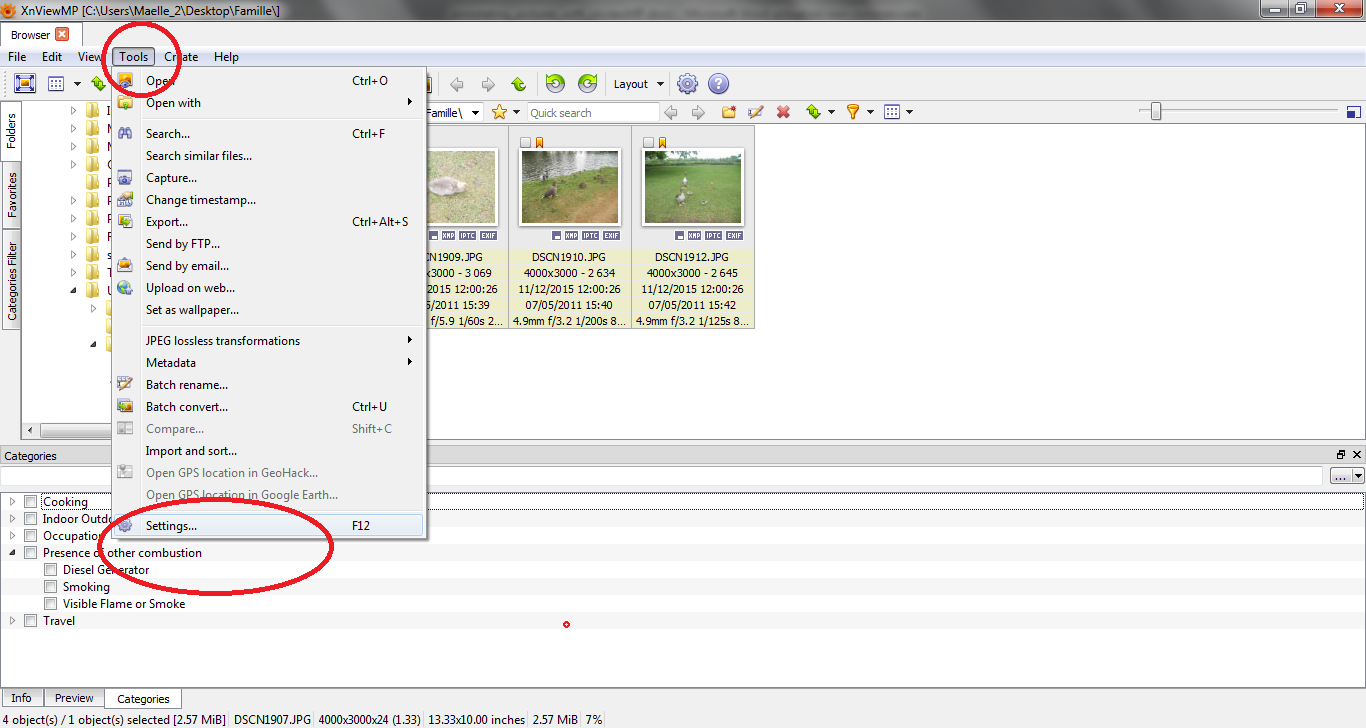


Then in the Metadata pane of the settings please check and uncheck the boxes so that it becomes exactly:


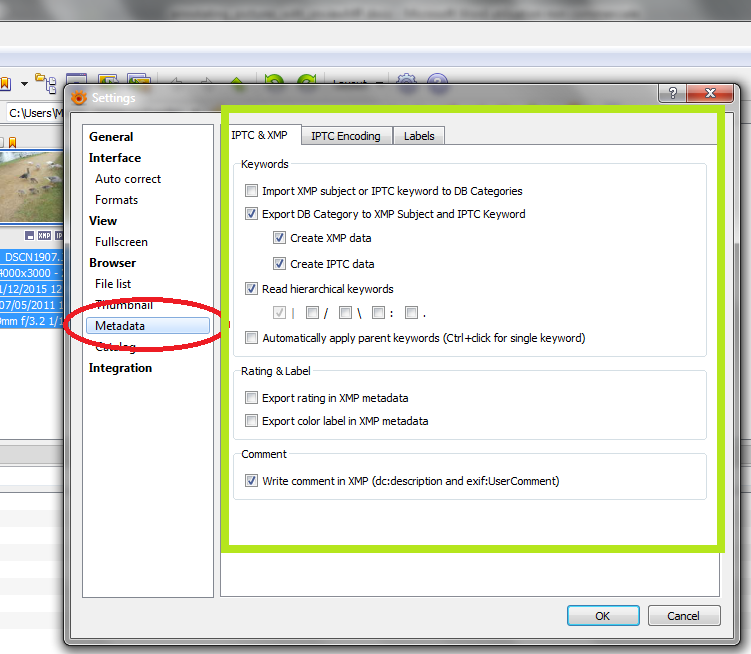


This means you have to check: “Export DB Category to XMP Subject and IPTC Keyword” and “Create XMP data” and “Create IPTC data” and “Read hierarchical keywords”.

You have to UNCHECK “Automatically apply parent keywords”.

## 4.3.4 Changing the layout

While coding, you’ll need to use the category pane, the previewer pane (because the thumbnails might be too small) and the pane with the thumbnails. Under View/Layout, and also by moving the panes manually, you can change the layout and get the one that works best for you. Take time setting it up, since it can improve your user experience.

You can also change the size of the thumbnails, see below (red for changing the size, purple for the zoom).


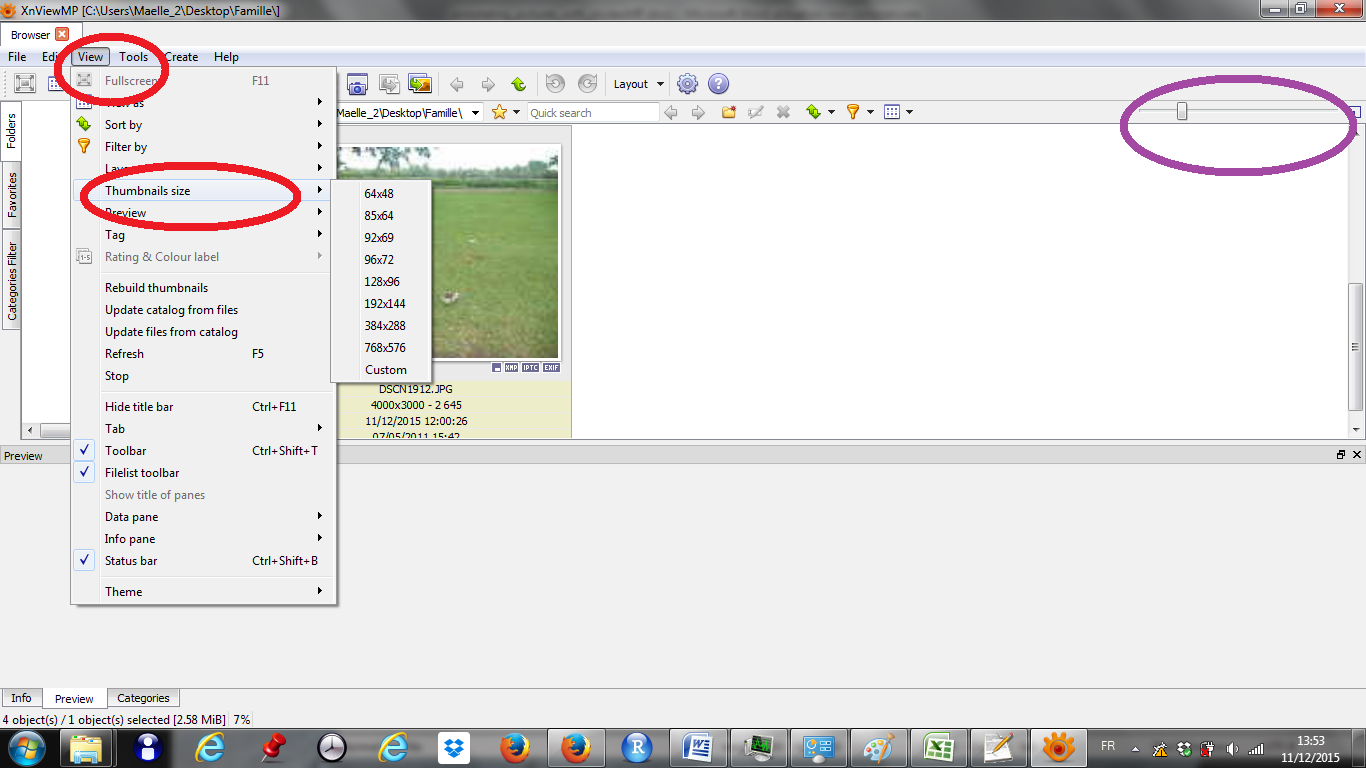


Afterwards, please close the software and open it again.

# 5. BROWSER TRAINING

**5.1 Browser Access**

- Open the xnviewMP Browser by clicking on the “XnViewMP**”** Icon on the desktop


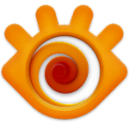


Work in a folder with a COPY of the photos, so that you keep the original photos in another folder. Use the folder structure on the left to open the folder containing the photos to be annotated:


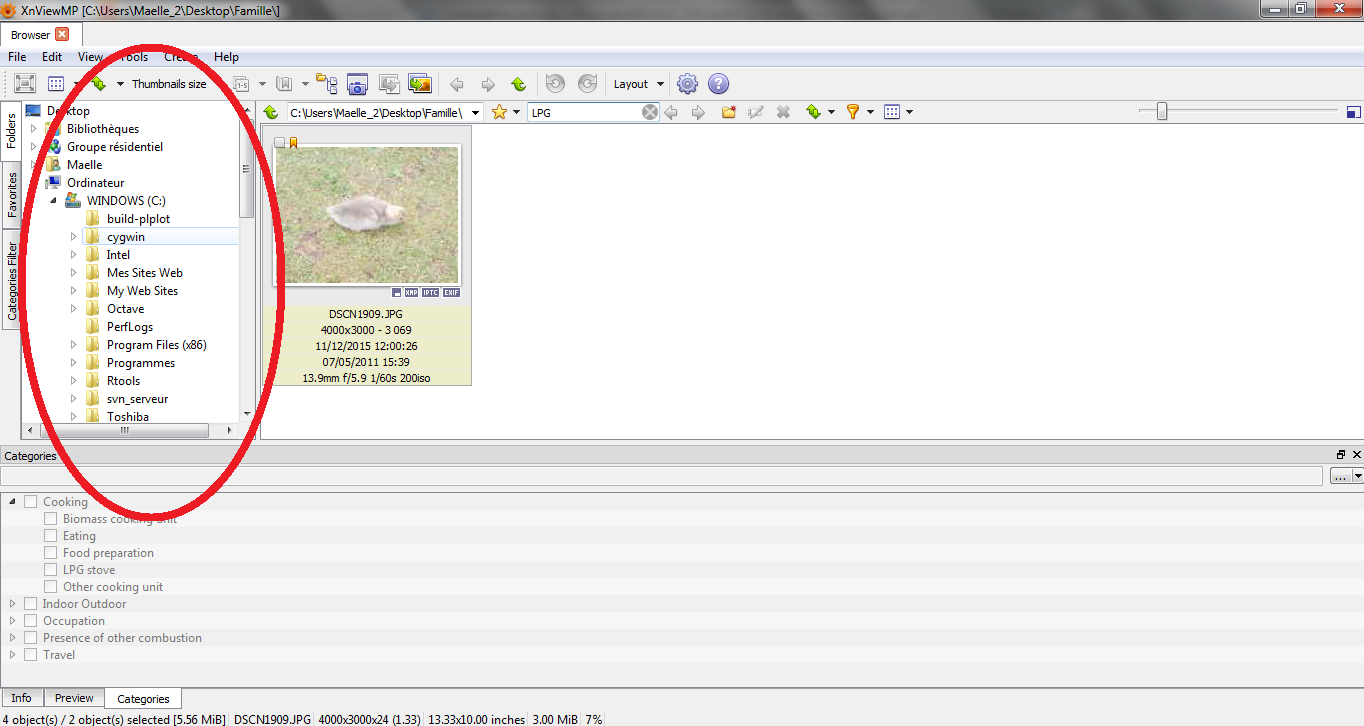


## 5.2 Adding annotations

You can select one or several photos, and then in the categories tab you can check the boxes corresponding to the code you wish to add. Please do not check the category one (i.e. do not check cooking, only check the codes of cooking that apply to the photo(s)).


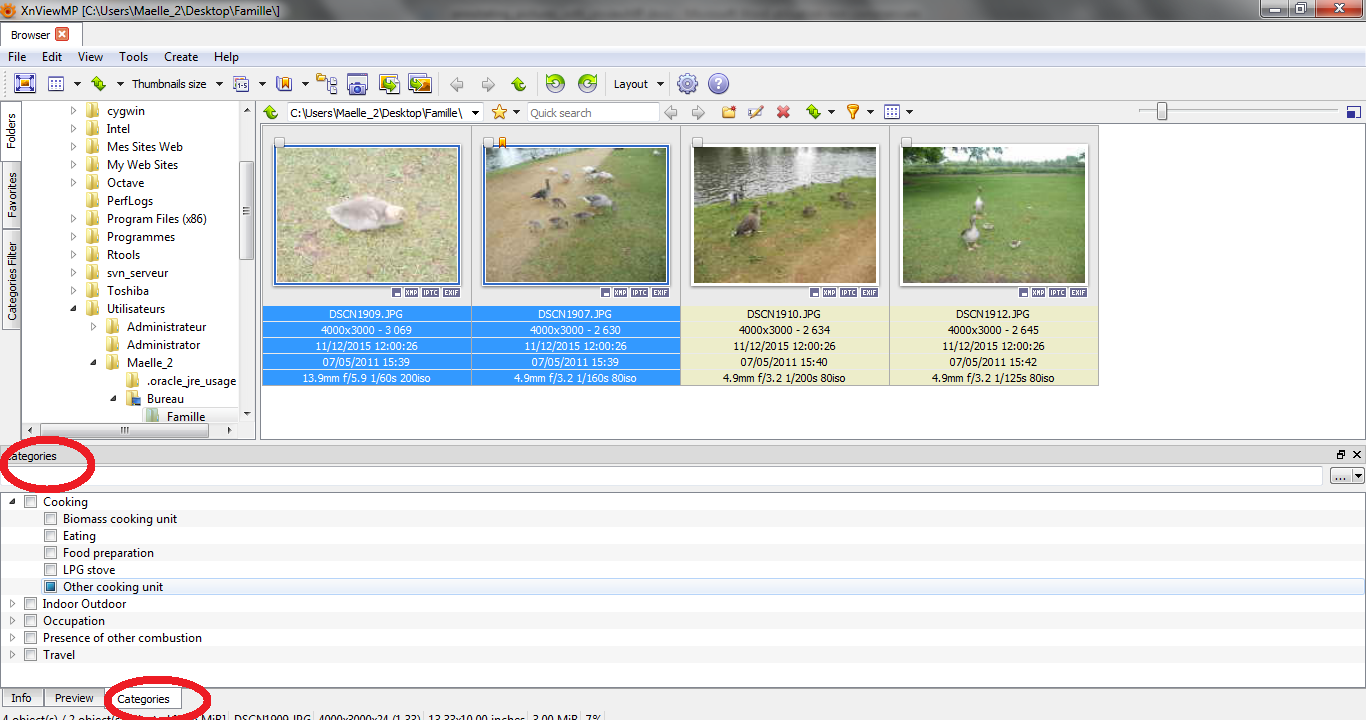


Do this for all the codes you want. Once you’re done you can filter photos according to a code and look at the photos that have this code.


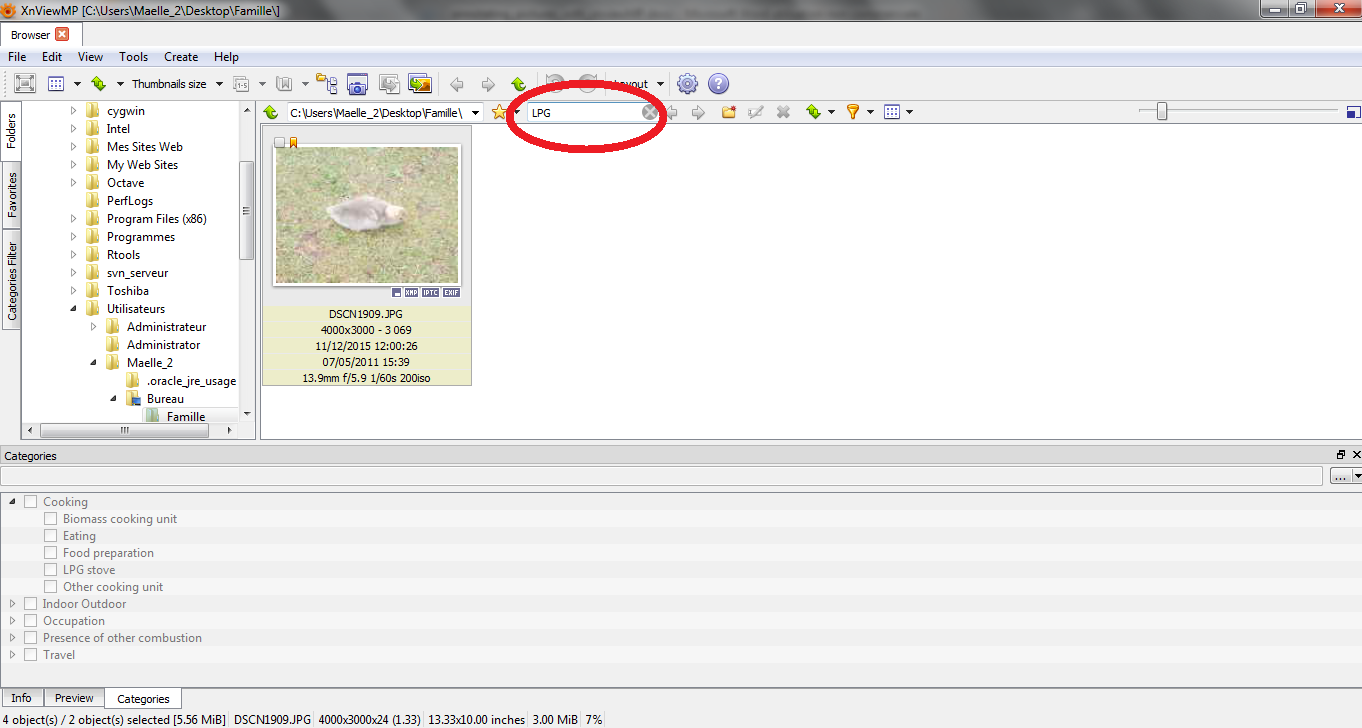


## 5.3 Exporting the annotations

At the end of a coding session you need to export the image-level annotations.

**FIRST SELECT ALL PHOTOS!!!**

Then click on Create/File listing


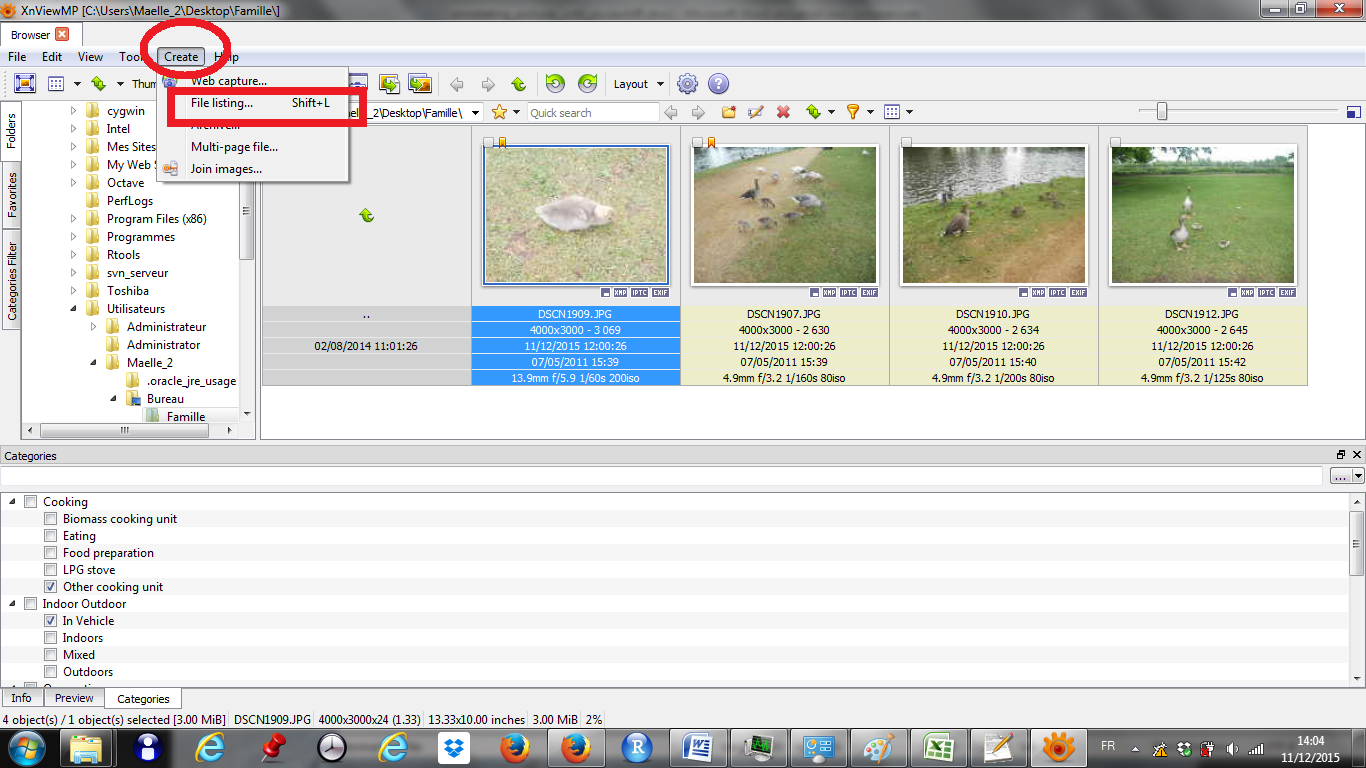


You need three variables in the export.

First select filename to get the filename of each photo.


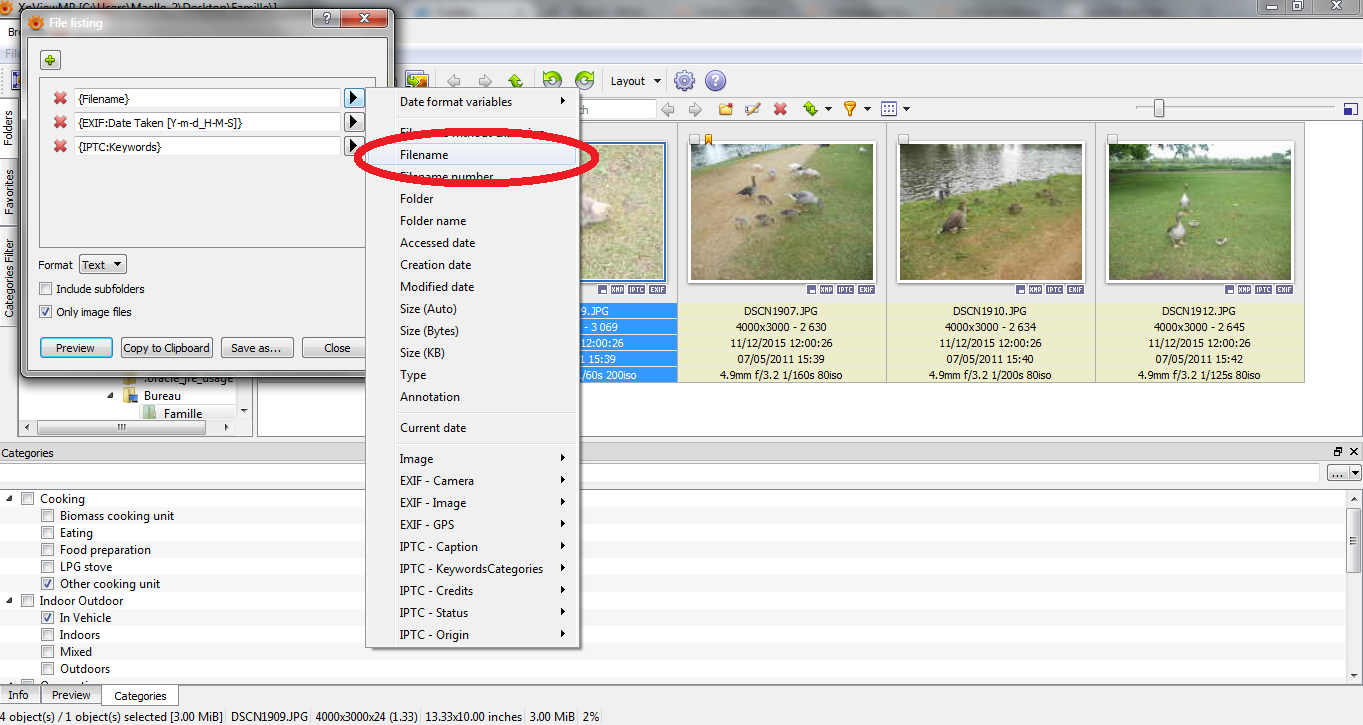


Then you need the creation date.


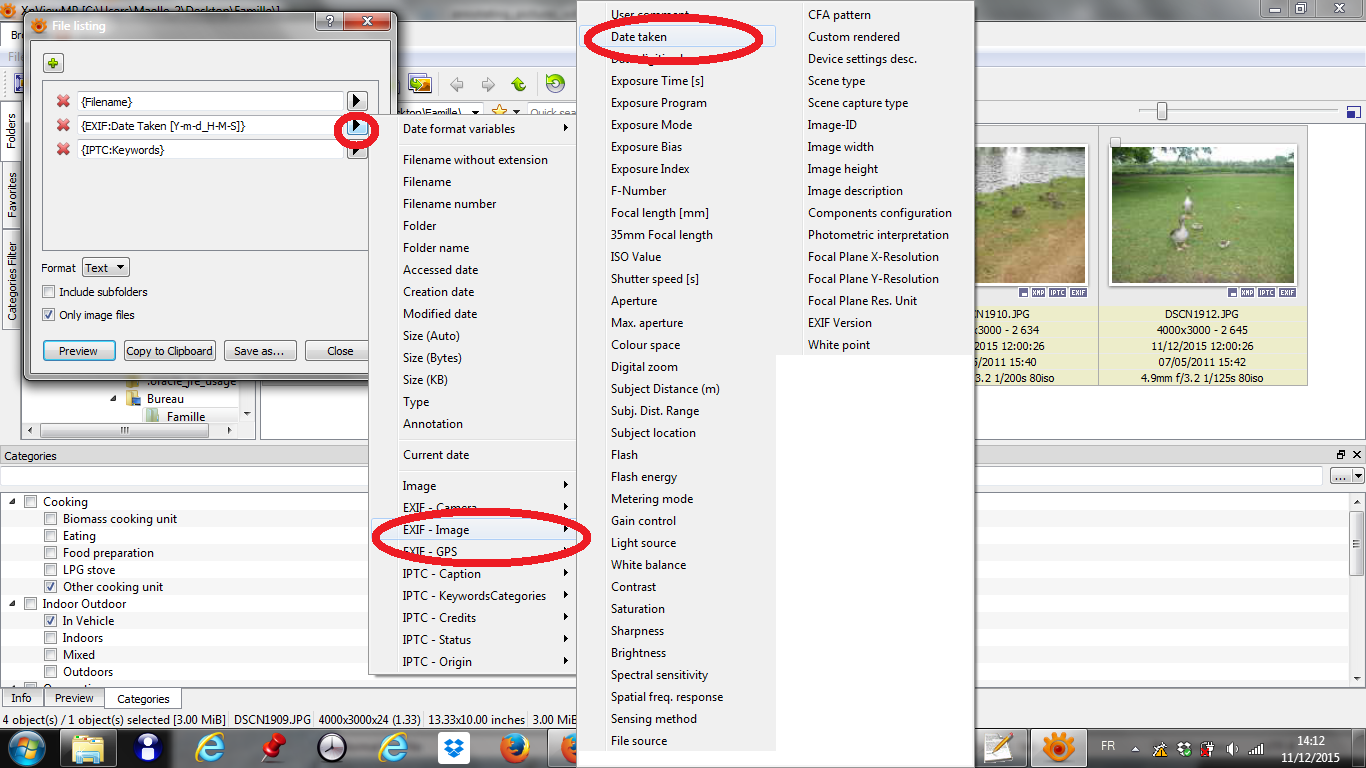


And finally you need the codes.


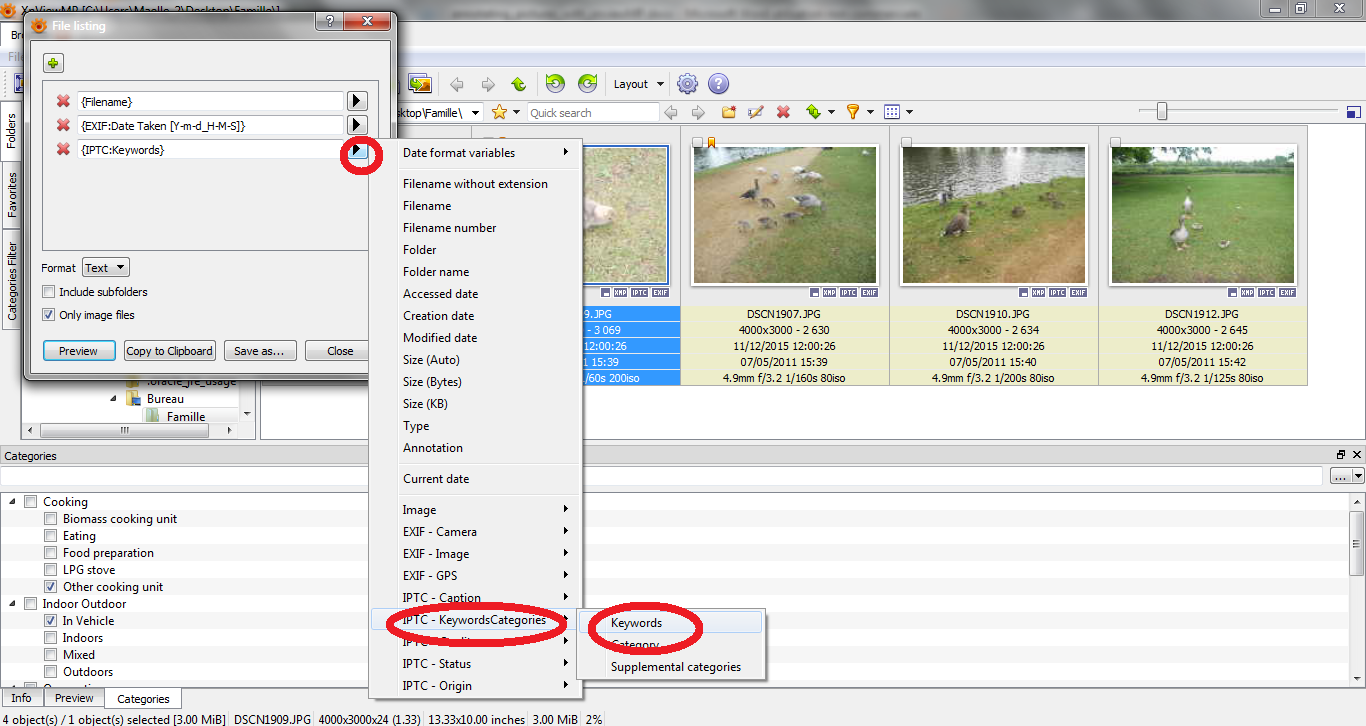


So this is what you should see. Choose the txt output format.


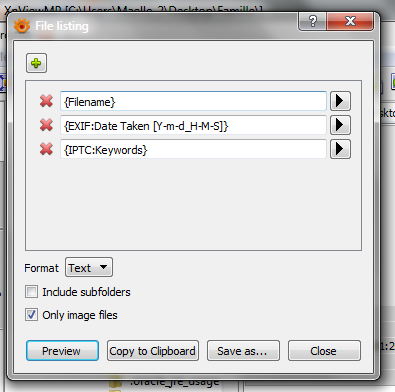


**Saving the exported file:**

Click save as… and select the location you would like to save the exported file to (we recommend one folder up from where the copy of the images are stored). And then save it with a name respecting the naming convention.

The file name should be in the following format VN_PN_SN_DDMMYY_AG_<device label>_Coding_PassCode_<Annotator S.No>

- - Example: - 21_12345_01_200415_AG_A_Coding_CK_01
    - Here VN is the village code
    - PN is the participant id
    - SN is the sample number (1 to 9)
    - DDMMYY is the sample start date
    - AG is autographer
    - device label is which autographer was used (A to F)
    - Coding says that this file has image coding data
    - Pass Code can be Cooking =CK, Travel=TP, Occupation=OP, Presence of other combustion=PM, Indoor/Outdoor=IO; Each pass should have a separate file.
    - Annotator S.No is the person who coded this data (e.g. 01, 02, 03…)

Open the export file to check if all photos have been exported (by checking against the total number of photos to the number of rows in the exported file) and have codes and that it looks good.

# 6. PROTOCOL TRAINING

#

The following pages can be printed and used as a reference during the coding process.

6.1 General Guidelines

### 6.1.1 Coding Photos

- - All consecutive images of an activity or environment following the last calibration photo (6.1.2) indicates the START of a session.
  - Depending on the pass, every image will be checked for the presence of category and sub categories.
- The 50% Rule:
  - Some images require subjective judgments by the annotator regarding the activity or environment. When an image cannot be coded using a specified decision tree or there is some uncertainty about what is occurring, we use the 50% rule.

▪ The 50% rule specifies that an ambiguous photo can still be coded if the annotator subjectively determines that the likelihood of something being present (i.e. the activity or environment in the image) is greater than 50%.

- - The 50% rule is NOT meant not as a precise calculation of probability but as a decision heuristic to guide difficult coding decisions. A subjective determination that something has a ≥ 50% of being present should be based on a judgment call that something is more likely than not to be present. The annotator can use any information present in the image or in adjacent images to determine whether something is more likely than not to be present. The annotator does NOT need to be very certain or confident in a code, only that it is more likely than not to be present.

6.1.2 Calibration Photos

Autographer images are intended to provide detailed and objectively measured time activity data. In order to perform a time based analysis on data, the autographer will be calibrated against a computer clock before start of each session.


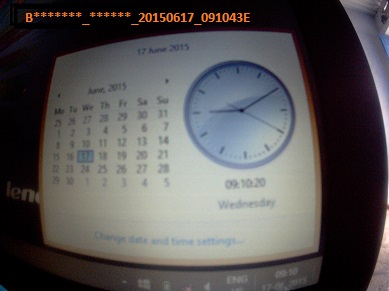


The next consecutive image to this calibration image will be the first photo of the session, where you can see the activity or the environment.

### 6.1.3 General Codes

The following are general codes that may be applied for any given pass.

| Code |  |
| --- | --- |
| **UNCODABLE** | If **any image is blocked** that image is considered **“uncodable”.** The image / images maybe even partially blocked, but may cause the annotator to miss a change in activity or environment.  If the last clear image and the first clear image are in the same context **it can be coded** (possibly using the 50% rule). |

## 6.2 Passes

Multiple passes at the data make Autographer coding much easier and more reliable. If the annotator needs to look for too many things in one pass they are more likely to miss something. Further, coding may be split up across annotator for example someone may have better activity coding skills than environment coding skills, employing these specialties in different passes is recommended.

For our current purposes, we will code data in 5 separate passes according to distinct categories, including: “Cooking,” “Transport,” “Occupation”, “Presence of other combustion” and “Indoor/Outdoor.”

| Category | **Pass** |
| --- | --- |
| Cooking | **CK** |
| Transport | **TP** |
| Occupation | **OP** |
| Presence of other combustion | **PM** |
| Indoor/outdoor | **IO** |

### 6.2.1 PASS 1: Cooking

Code the images into categories based on ***cooking unit visible in each image (type of fuel), irrespective of its status (active or inactive).***

Code the images using the following codes for Cooking.

Coding is non-exclusive and multiple codes may be given to single event. Ex: Event 1: Presence of biomass cooking unit and LPG stove at same time (A,B).

However separate images into events if one follows the other Ex: presence of biomass cooking unit in 20 images (A), followed by presence of LPG stove in 10 images (B).

| **Code (01)** | **Examples** |
| --- | --- |
| - 1. Biomass cooking unit   2. LPG stove   3. Other cooking unit   4. Food preparation   5. Eating   6. Presence in the kitchen | Type of fuel (visual cue):   - Biomass cooking unit (fuel- wood, dung, grass/leaves) - LPG stove- gas stove - Other cooking unit- table top kerosene stove with piston, not able to identify fuel type. - Food preparation – Mostly before, during and after cooking- Involves cleaning/ chopping vegetables, washing or holding dishes , moving in kitchen - Eating – eating or drinking – utensils with food served is visible - Presence in kitchen – Any cooking unit or utensils visible in the surroundings |

### 6.2.2 PASS 2: Travel

Code the images into categories based on ***participant being present on a road, irrespective of presence of traffic***. Road is a path meant for movement of people or vehicles and includes the adjacent area (outdoor only) on both sides. Adjacent areas can be veranda, drive way, sidewalk, bus-stand etc. Exclude front & back yard of buildings which maybe gated or walled.

Apply codes provided for specified travel modes - bus, car, bicycle, auto, motorcycle (A-E). Else, apply code (F) -present on road.

Code the images using the following codes for travel

Coding is mutually exclusive. Ex: travelling by bicycle on a road 20 images (C), followed by sitting on road 10 images (F).

| **Code (02)** | **Examples** |
| --- | --- |
| **A. Travel by Bus**  **B. Travel by Car**  **C. Travel by Bicycle**  **D. Travel by Auto-rickshaw**  **E. Travel by Motorcycle**  **F. Participant present on road** | Visual cue:  If the participant is travelling using specified travel-modes, apply that code: bus, car, bicycle, auto, motorcycle. Code travel mode used by the wearer, even if he/she is temporarily stationary.  Visual cues can be handlebars of a bicycle or motorbike; dashboard/steering wheel/seats for a car; inside photo of a bus or auto;  If participant is present on road, but not travelling using specified mediums (A-E), code as (H)- present on road. Ex: walking, sitting, standing, pushing a cart, running, on bullock cart or tractor etc. |

### 6.2.3 PASS 3: Occupation

Code the images into categories based on occupation i.e.

- ***participant present at a formal worksite*** (worksite type)- (A,B,C)

**We apply the code, irrespective of any activity or non-activity of the person (ex: subject travelling within in a work-site)*

OR

- ***participant working at an informal worksite or other setting*** -(D)

Code the images using the following codes for occupation. Codes are mutually exclusive.

| **Code (03)** | **Examples** |
| --- | --- |
| 1. Present at formal worksite (enclosed area -office, shop) 2. Present at formal worksite (field) 3. Present at formal worksite ( industry /factory) 4. Working at informal worksite or other setting | Visual cues: wearer in same setting for prolonged period of time, involved in work (set of tasks) related to occupation (farming, selling goods in a shop etc.). Visible work-related processes /goods/instruments etc.   - present at formal worksite(enclosed area- office,shop): presence of computers, desks, books, inside a shop outlet. It should be a enclosed area - present at formal worksite(field) : agricultural area (fields), tools - present at formal worksite (industry/factory) : industrial machines or processes, construction site - working at informal worksite or other setting: not in an formal worksite (A-C): enclosed area (office,shop), field (agriculture), industry (factory) Ex: street vendor, basket weaving on road, tailoring at home etc. |

### 6.2.4 PASS 4: Presence of other combustion

Code the images into categories based on ***visible source of combustion irrespective of it being active*** (diesel generator or cigarette) OR ***Visible flame or smoke*** (big or small)

Code the images using the following codes for combustion.

Codes are non-exclusive and multiple codes can be assigned to single event. Ex: Smoking cigarette and being near a diesel generator (A,B)

However create separate events if one is followed by the other. Ex: Smoking cigarette and near a diesel generator in 6 images, followed by smoking cigarette in 5 images. This will be Event 1: Smoking cigarette and near a diesel generator in 6 images (A,B), followed by Event 2: smoking cigarette in 5 images (B)

| **Code (04)** | **Examples** |
| --- | --- |
| 1. Diesel generator 2. Smoking 3. Visible flame or smoke | Visual cue:   - Diesel generator: generator in view - Cigarette smoking: Includes active and passive smoking. Cigarette visible in hand or mouth of participant or other individuals. For each such image: Code 2 images to the right, image with cigarette, & 2 images to the left to standardize duration of smoking. - Visible flame or smoke - flame (fire) or smoke. Ex: trash burning, agricultural produce, cremation, tyre burning, brick kiln or rice husk, match-stick, kerosene lamp, Diya |

### 6.2.5. Pass 5: Indoor/Outdoor Pass

Sort the images based on the ***environment of the wearer***. *All images should be coded by one of the codes below i.e. there would be no image without an indoor/outdoor code, unless it is ‘uncodable or ‘not sure’*

| **Code 04** |  |
| --- | --- |
| A. **INDOORS** | Visual Cue: at least three walls and a roof (eg. garage). The walls need not touch the roof (ex: cellar). “Half-walls” such as those enclosing a balcony are considered as walls and should be coded as “Indoors”  Include: Parking garages, balconies, apartment complex corridors |
| **B. OUTDOORS** | Visual Cue: Sky, trees, pavement . This includes travel by bike or motorcycle. |
| **C. IN VEHICLE** | Includes: Riding in a car, bus, trolley, train or any other vehicle. Travel by bike or motorcycle is not part of this code. It is to be coded as “outdoors” |
| **D. MIXED** | If participant is midway between indoor and outdoor for example in the doorway and cannot make a judgement of the context of what the participant is transitioning into to determine the code [e.g. opening a door to go indoors would be coded as Indoors] |

**7. List of codes**

**Uncodable**

**Cooking (CK)**

Biomass cooking unit

LPG stove

Other cooking unit

Food preparation

Eating

Presence in the kitchen

**Travel (TP)**

Travel by bus

Travel by bicycle

Travel by auto-rickshaw

Travel by motorcycle

Travel by car

Participant presence on road

**Occupation(OP)**

Presence at Office or Shop

Presence at Work Field

Presence in Industry

Presence in Informal Work

**Presence of other combustion(PM)**

Diesel Generator

Smoking

Visible Flame or Smoke

**Indoor Outdoor(IO)**

Indoors

Outdoors

In Vehicle

Mixed

#

**8. Example Images**

**GENERAL**

**Uncodable**


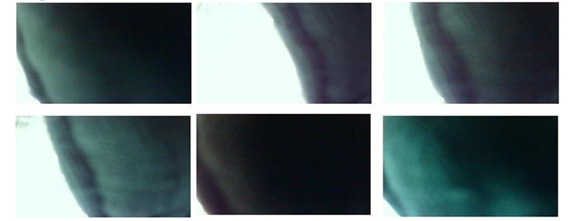


**COOKING**

| Biomass cooking unit | LPG stove | Other cooking-units |
| --- | --- | --- |
| 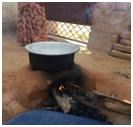 | 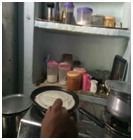 | 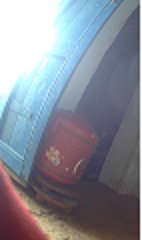 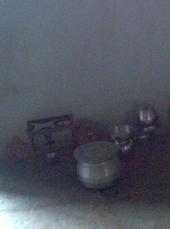 |

| Eating | Presence in the kitchen | Food Preparation |
| --- | --- | --- |
| 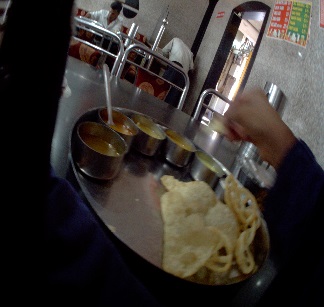 | 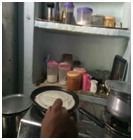 | 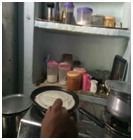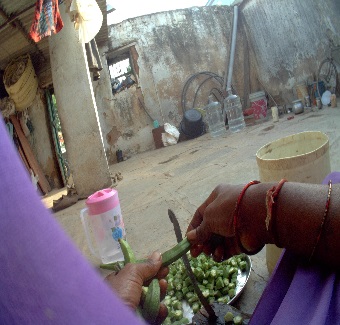 |

**TRAVEL**

Travel by bus


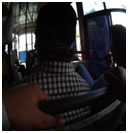

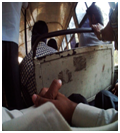

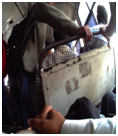

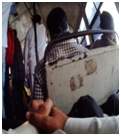

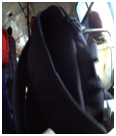


Travel by car


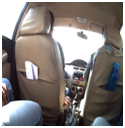

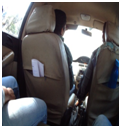

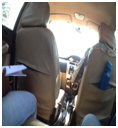

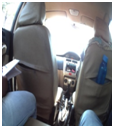

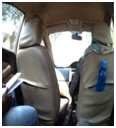


Travel by bicycle


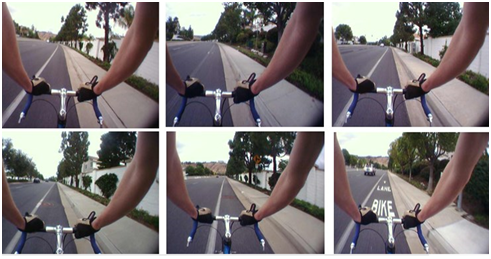


Travel by auto-rickshaw


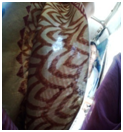

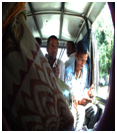

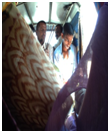

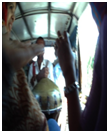


Travel by motorcycle


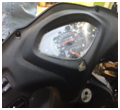

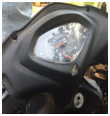

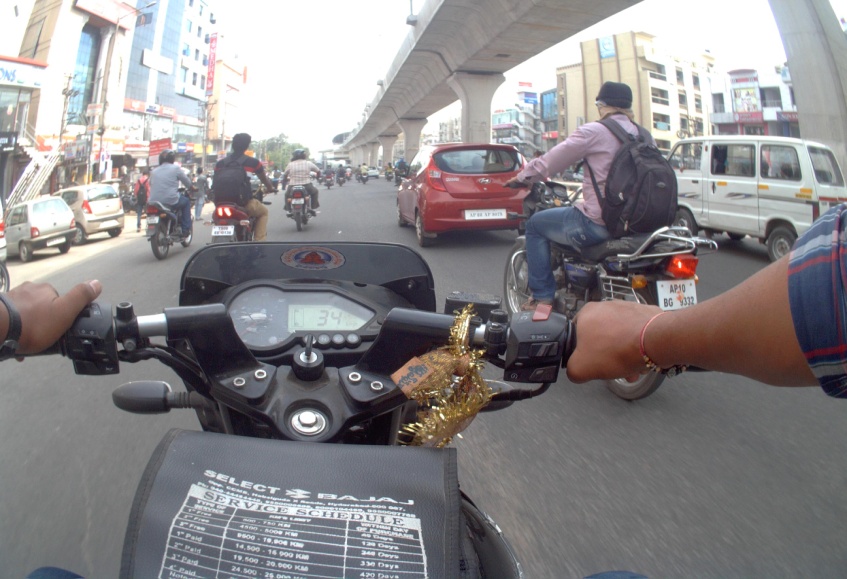


Participant present on road


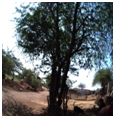

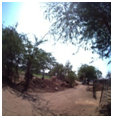

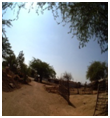

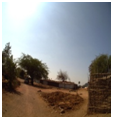

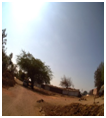


**OCCUPATION**

Present at formal work-site: enclosed area (shop, office)


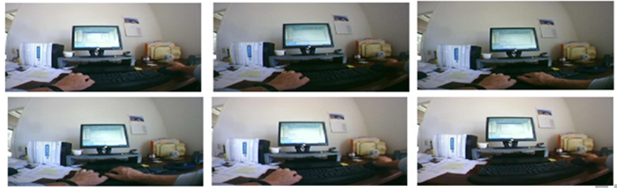


Present at formal work-site: field (agriculture)


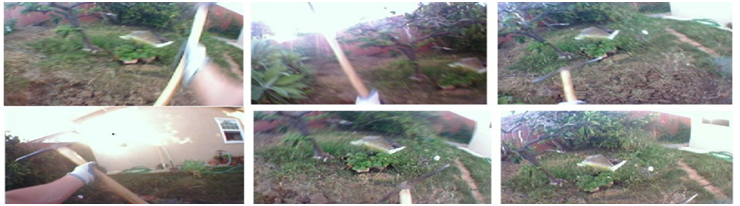


Present at formal work-site: industry (factory)


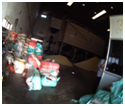

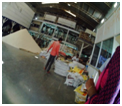

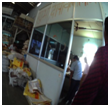

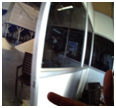


Working in informal work-site or other setting

|  |  |  |  |
| --- | --- | --- | --- |


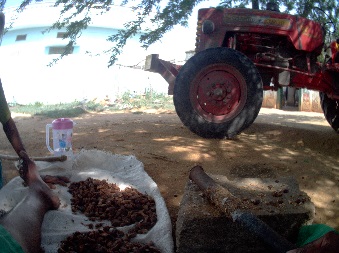

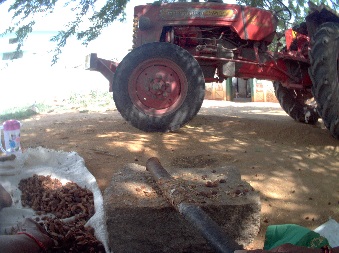

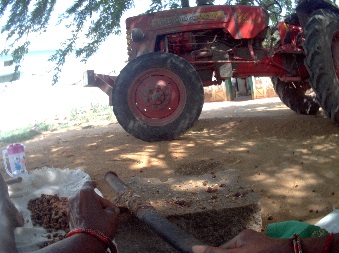

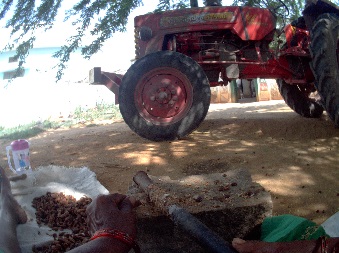


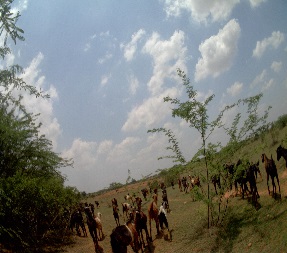

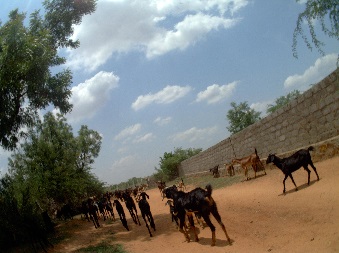

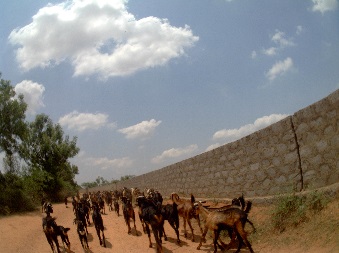

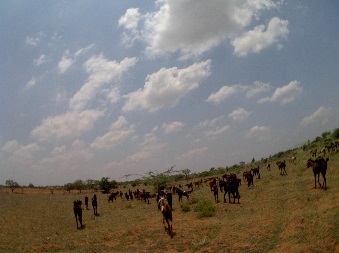


**PRESENCE OF OTHER COMBUSTION**

Presence of nearby diesel generator


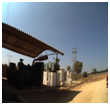

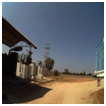

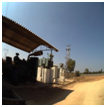

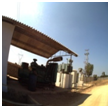

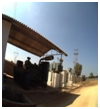


Cigarette smoking


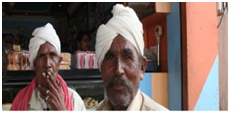


Visible smoke or flame (trash burning)


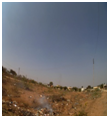

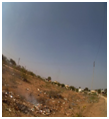

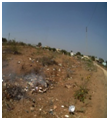

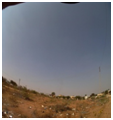

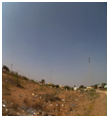


**INDOOR/OUTDOOR**

Indoor


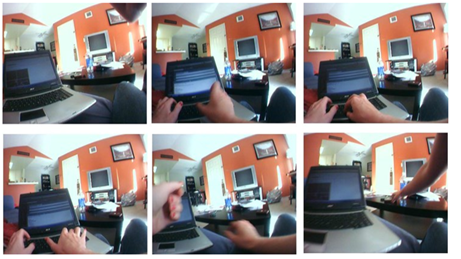


Outdoor


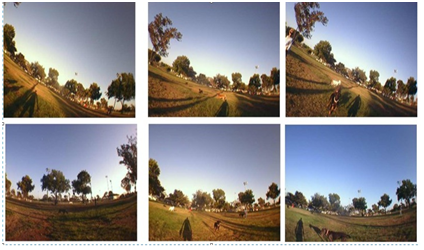


In Vehicle


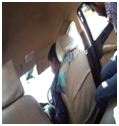


Mixed


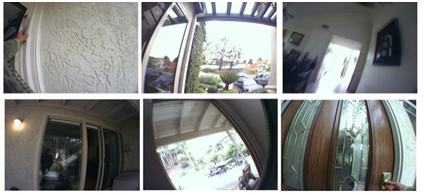

Supplement: Supplementary file 1 — Supplementary material [file mmc1.docx]
